# Exploring Mechanistic Targets of *Areca catechu* Against Neurodegenerative Diseases Through an Integrated Network Pharmacology, Molecular Docking, and Experimental Approaches

**DOI:** 10.3390/ijms27125169

**Published:** 2026-06-07

**Authors:** Sakawrat Janpaijit, Kanika Verma, Ansella Amanda Epifani Widoyanti, Tewin Tencomnao, Anchalee Prasansuklab

**Affiliations:** 1 College of Public Health Sciences, Chulalongkorn University, Bangkok 10330, Thailand; sakawrat.j@chula.ac.th (S.J.); ansellaamanda@gmail.com (A.A.E.W.); 2 Center of Excellence on Natural Products for Neuroprotection and Anti-Ageing (Neur-Age NatChula), Chulalongkorn University, Bangkok 10330, Thailand; tewin.t@chula.ac.th; 3 Department of Molecular Epidemiology, ICMR-National Institute of Malaria Research, New Delhi 110077, India; kanika.honey.verma@gmail.com; 4 Department of Clinical Chemistry, Faculty of Allied Health Sciences, Chulalongkorn University, Bangkok 10330, Thailand

**Keywords:** areca nut, Alzheimer’s disease, Parkinson’s disease, therapeutic mechanisms, neuroinflammation, microglia, *Caenorhabditis elegans*

## Abstract

Alzheimer’s disease (AD) and Parkinson’s disease (PD) are the two most prevalent neurodegenerative disorders, while the therapeutic efficacy of current drugs for both diseases remains limited, with unfavourable side effects. The fruit of *Areca catechu* L. (AC) is recognised as a popular chewing item across China and Southeast Asia and has been used for centuries as a traditional remedy, ranging from relieving digestive issues to depression. The neuroprotective role of AC has been underscored in previous studies; however, its mechanisms of action remain unclear. The present study aimed to investigate anti-neurodegenerative mechanisms of AC for the treatment of AD and PD. An integrated approach combining untargeted metabolite profiling, network pharmacology, bioinformatics analysis, and molecular docking was utilised. Experimental validation was performed using in vitro cell-based and in vivo models. The study revealed TNF-α, IL-1β, IL-6, CASP3, MAPK3, and AKT1 as top-ranked hub targets by which AC exerts its action on AD and PD. Enrichment analyses of these genes identified significant biological and functional pathways involved in neuroinflammation, apoptosis, and AD. Experimental validation showed that AC extracts significantly downregulated hub gene expressions in the neuroinflammatory BV-2 microglia cell model and prolonged the survival of the transgenic *Caenorhabditis elegans* AD model. Docking analysis suggested lucidine B, oxolucidine B, solanocapsine, evodiamine, and liquiritigenin are the principal phytocompounds underlying the neuroprotective properties of AC. The findings revealed the pharmacological mechanisms of AC and highlighted its potential value as an effective, multitargeting natural agent to address challenges in AD and PD therapies.

## 1. Introduction

Due to global demographic shifts, particularly population ageing, neurodegenerative disorders (NDDs) have posed a significant challenge and burden for healthcare systems and global public health. NDDs commonly refer to a group of neurological illnesses, including Alzheimer’s disease (AD), Parkinson’s disease (PD), multiple sclerosis, amyotrophic lateral sclerosis, and Huntington’s disease, which are characterised by the progressive structural and functional decline of neurons, leading to neuronal cell damage and death [1]. Among them, AD and PD are the first and second most prevalent types of NDDs. AD also represents the predominant form of dementia, accounting for approximately 60% to 80% of cases globally. With ageing as the primary risk factor, the number of individuals living with AD and PD is projected to rise rapidly to over 130 million by 2050 and 17 million by 2040, respectively [2,3]. Generally, the pathophysiological characterisation of AD involves extracellular deposition of amyloid-beta (Aβ) plaques and intracellular deposition of hyperphosphorylated Tau as neurofibrillary tangles (NFTs). Both abnormal protein aggregates trigger several neurotoxic events, including oxidative stress, mitochondrial dysfunction, neuroinflammation, impaired neuronal function, disrupted synaptic plasticity, and cell apoptosis [4]. While the primary affected brain region in AD is the hippocampus, the pathological features of PD involve the loss of dopamine-producing neurons in the substantia nigra (SN), caused by intraneuronal inclusions of α-synuclein (α-syn) that form Lewy bodies (LBs) and Lewy neurites (LNs) [5]. While a substantial increase in AD and PD patients is estimated, it must also be noted that current medications for both diseases have limited effectiveness, as they primarily provide temporary symptomatic relief without altering disease progression and are often associated with unfavourable effects [6]. Therefore, searching for new, effective therapeutics is considered urgent and critical. In this context, extracts and phytocompounds derived from natural sources are of high interest as promising drug candidates with multi-beneficial bioactivities for neuroprotection [7].

The fruit of *Areca catechu* L. (AC), also known as areca nut, is recognised as a popular chewing item and long-used traditional herbal remedy across China and Southeast Asia. This plant belongs to the Arecaceae family of palms and is cultivated in tropical regions [8]. It also holds significance in cultural rituals in several Southeast Asian countries. The tradition of chewing betel quid, a mixture comprising AC, betel leaf, slaked lime, and sometimes tobacco, has been deeply rooted in social and cultural practices of local communities for thousands of years. Additionally, AC has played an important role in traditional herbal remedies such as Ayurveda, traditional Chinese medicine, and Thai traditional medicine for the treatment of various illnesses, ranging from digestive issues and parasitic diseases to wound healing, fever, and depression [9,10]. This plant contains many bioactive constituents, including polysaccharides, polyphenols, flavonoids, fatty acids, and, in particular, alkaloids, which are found highly abundant in the fruit part. These active compounds offer a diverse array of pharmacological properties, for instance, antioxidant, anti-inflammatory, immunomodulatory, anti-microbial, anti-helminth, anti-depressant, analgesic, anti-cancer, anti-acetylcholinesterase, digestive stimulating, anti-diabetic and lipid-lowering effects [11,12,13,14,15]. Moreover, our recent study revealed that AC ethanolic extract and its derived compounds could attenuate anthracene-induced neuroinflammation and oxidative damage in BV-2 microglial cells [16]. While several previous and recent studies have underscored the neuroprotective role of AC, its potential mechanisms of action in AD and PD therapies remain unclear. Thus, this study aimed to explore the potential anti-neurodegenerative mechanisms of AC for the treatment of AD and PD.

In modern natural product-based drug discovery, network pharmacology is a powerful, interdisciplinary, systematic approach used to explore and elucidate the mechanisms underlying the therapeutic effects of natural extracts against various diseases [17]. This approach combines bioinformatics, system biology, and computational modelling with pharmacology to analyse the complex interactions between phytochemical components and specific molecular targets within biological networks and pathways relevant to a disease of interest. It also helps provide valuable insights into the therapeutic actions of crude extracts, which result from the cumulative and synergistic effects of multiple constituents in a mixture acting on multiple targets [18]. The present study utilised network pharmacology in combination with molecular docking, in vitro cell and in vivo animal experiments, to investigate the AC’s active constituents and their relevant molecular targets and pathways of action against AD and PD. In the study, the active phytochemicals in AC extracts, identified through untargeted metabolite profiling, and their corresponding disease-related targets were screened using network pharmacology and subsequently used to construct the compound–target–disease networks. Further, the top targets in biological networks were selected to verify their interactions with AC’s active constituents using molecular docking, followed by experimental validation of target genes’ expression changes in BV-2 microglial cells. Additionally, the protective effect of AC extracts was examined in a *Caenorhabditis elegans* (*C. elegans*) model of AD.

## 2. Results

### 2.1. Potential Active Phytocompounds in AC Extracts and Their Associated Targets

**Figure 1:**
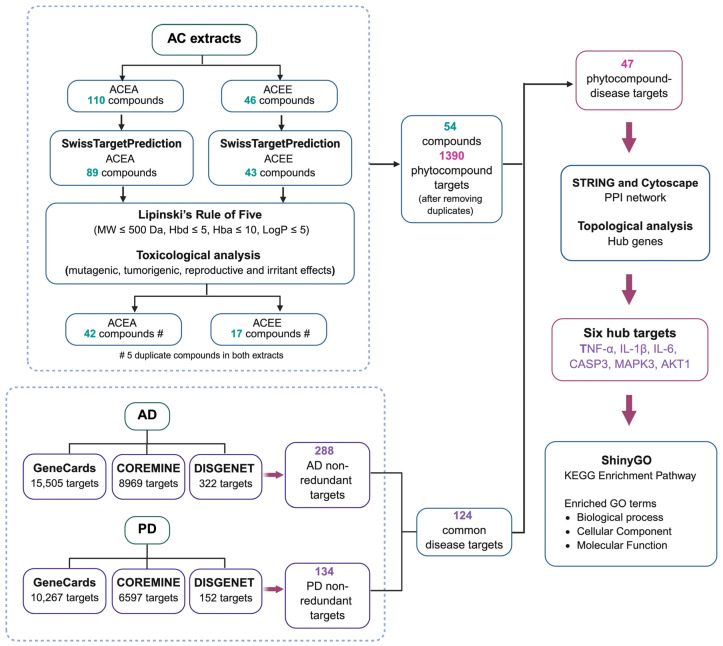
The workflow for network pharmacology analysis of AC extracts against AD and PD. AC, Areca nut; ACEA, ethyl acetate extract of AC; ACEE, ethanolic extract of AC; AD, Alzheimer’s disease; PD, Parkinson’s disease; MW, molecular weight; Hbd, hydrogen bond donor; Hba, hydrogen bond acceptor; LogP, logarithm of the partition coefficient; PPI, protein–protein interaction; KEGG, Kyoto Encyclopedia of Genes and Genomes; GO, gene ontology.

A total of 110 and 46 phytocompounds in ACEA and ACEE, respectively, were tentatively identified through LC-QTOF analysis (see Supplementary Figures S1 and S2 and Supplementary Tables S1 and S2). The identified compounds were first screened for their associated target genes using the SwissTargetPrediction database. Then, the 89 compounds in ACEA and 43 compounds in ACEE showing their associated targets from prediction were subsequently evaluated for their drug-likeness and toxicological properties, which finally obtained 54 nonredundant phytocompounds from both AC extracts that fulfilled Lipinski’s Rule of Five and exhibited non-toxicological effects for further investigation. The lists of 54 phytocompounds, comprising 37 compounds in ACEA, 12 in ACEE, and five in both extracts, are shown in Table 1 and Table 2, respectively. The five duplicated compounds include (+)-eudesmin, buddledin A, montanol, podecdysone B, and quinic acid. From the selected 54 compounds, a total of 1390 phytocompound targets were successfully predicted and used in the subsequent analysis. The detailed workflow of the network pharmacological investigation in this study is summarised in Figure 1.

### 2.2. Identification of AD and PD-Related Targets and Phytocompounds–Disease Targets

**Figure 2:**
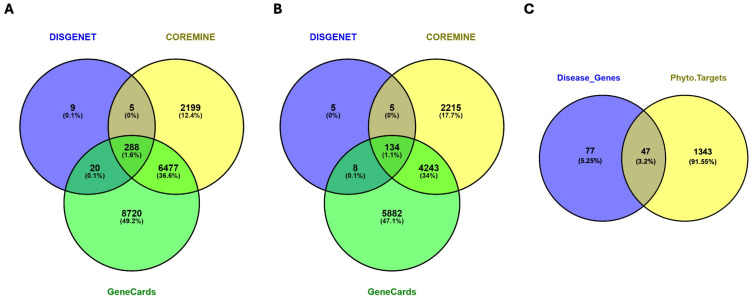
Venn diagram of the intersection targets. (**A**) The intersection of AD-related targets, (**B**) the intersection of PD-related targets, and (**C**) the intersection of AC phytocompound targets and common disease targets in both AD and PD.

The disease targets of AD and PD were identified from three databases: GeneCards, COREMINE, and DisGeNET. By using “Alzheimer’s” as a search keyword, the potential AD-related targets were retrieved, including 15,505 targets from GeneCards, 8969 targets from COREMINE, and 322 targets from DISGENET. Of these, the 288 shared targets among databases were identified (Figure 2A). In search of potential PD-related targets, “Parkinson’s” was used as a keyword. The results revealed 134 shared targets across three databases (10,267 from GeneCards, 6597 from COREMINE, and 152 from DisGeNET) (Figure 2B). Subsequently, the intersection analysis of 124 common disease targets shared by AD and PD with 1390 phytocompound targets identified 47 potential targets of AC extracts that are relevant to both diseases (Figure 2C). The list of AD-related targets, PD-related targets, and both diseases-related targets were shown in Supplementary Table S3.

### 2.3. PPI Network Construction and Topology Analysis of Hub Genes

**Figure 3:**
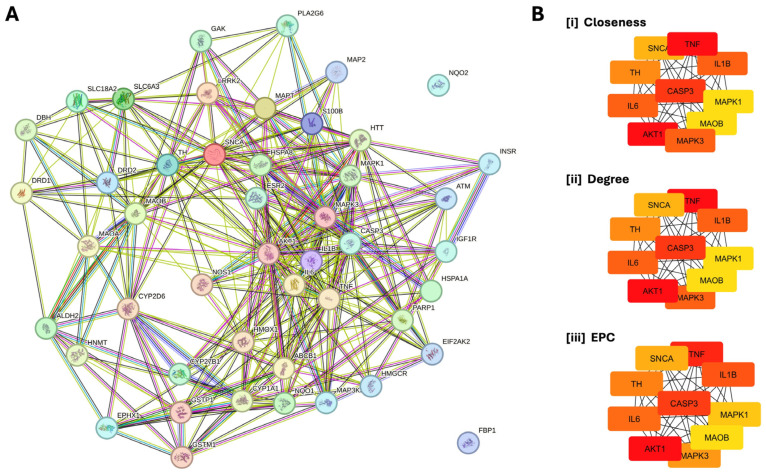
PPI network and topological analysis of the 47 targets correlated with phytocompounds in AC extracts. (**A**) STRING-based PPI network showing common targets. (**B**) Cytoscape visualisation of key targets identified by topological analysis using three algorithms, including (**i**) closeness centrality, (**ii**) degree centrality, and (**iii**) EPC.

To examine the functional relationships among the 47 identified phytocompound–disease targets, a PPI network was constructed and analysed for topological properties. The targets were imported into the STRING database and Cytoscape version 3.10.0 to construct and visualize the PPI network comprising 47 nodes connected by 330 edges, as shown in Figure 3A. The nodes represent individual targets, whereas the edges denote the interactions between them, providing an overview of the molecular connectivity among the identified 47 targets. Furthermore, network topology analysis was conducted to assess the structural significance of different targets in the PPI network. Node degree represents the number of direct interactions a given target has with other nodes and serves as a basic measure of network centrality. A node with higher values is often associated with greater connectivity and biological relevance (hub nodes). Accordingly, by performing topological analysis using three algorithms (closeness centrality, degree centrality, and EPC), the six hub targets (genes) including tumor necrosis factor-alpha (TNF-α), interleukin-1 beta (IL-1β), interleukin-6 (IL-6), caspase 3 (CASP3), mitogen-activated protein kinase 3 (MAPK3) and AKT serine/threonine kinase 1 (AKT1), were selected based on their connectivity scores as top-ranking nodes across all algorithms (Figure 3B). These nodes (hub targets) were then subjected to GO and KEGG pathway enrichment analyses to explore related biological processes, molecular functions, and cellular components, as well as metabolic and signaling pathways.

### 2.4. GO and KEGG Pathway Enrichment Analysis

**Figure 4:**
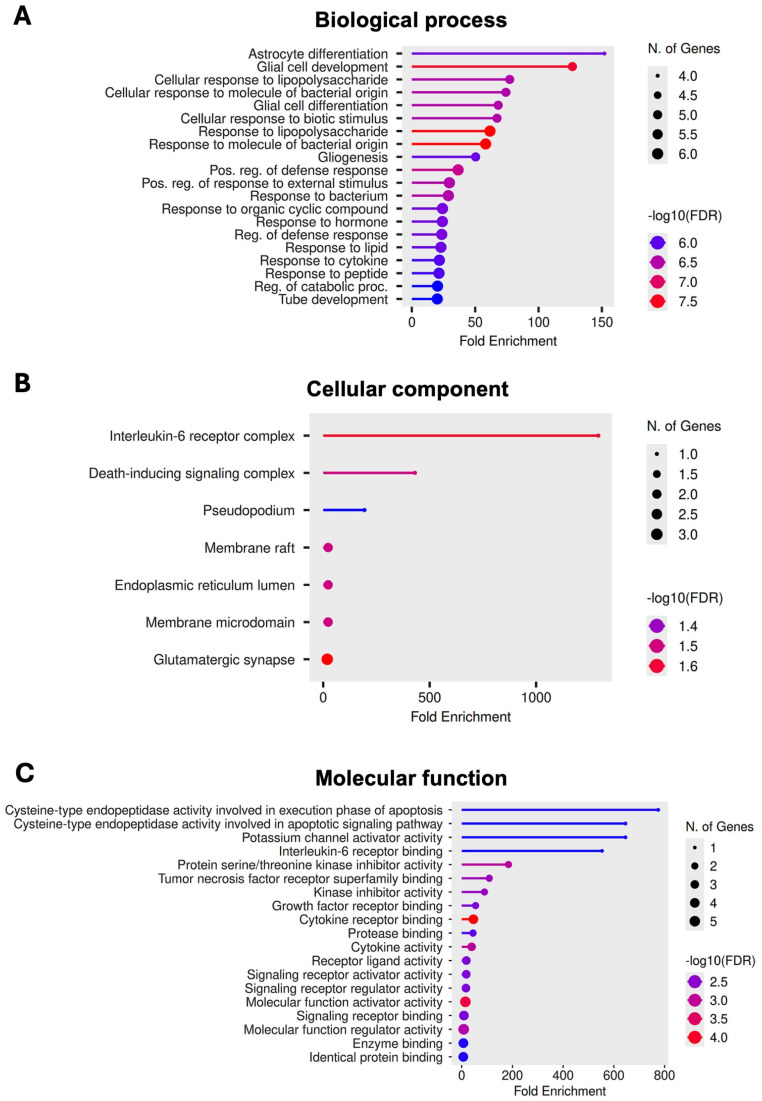
GO enrichment analysis of hub targets. Bubble plots of the top enriched GO terms of six hub targets in (**A**) Biological processes, (**B**) Cellular component, and (**C**) Molecular function. The *x*-axis shows the fold enrichment values, while the *y*-axis indicates the enriched GO terms. The bubble size represents the number of targets, and the colour indicates −log10(FDR) values.

**Figure 5:**
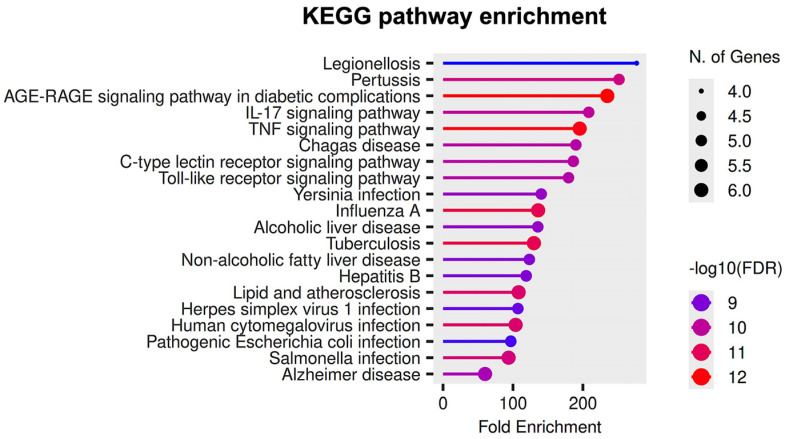
KEGG pathway enrichment and disease enrichment analysis of hub targets. Bubble plots showing the top 20 enriched KEGG pathways of six hub targets. The *x*-axis shows the fold enrichment values, while the *y*-axis indicates the enriched KEGG pathways. The bubble size represents the number of targets, and the colour indicates −log10(FDR) values.

The GO and KEGG pathway enrichment analyses were performed on the six hub genes using ShinyGo 0.85 to further elucidate the functional roles of AC phytocompounds in relation to AD and PD. The classification results for the top significant overrepresented GO terms, based on -log10 false discovery rate (FDR) values less than 0.05, across the biological process, cellular component, and molecular function categories, are shown in Figure 4. In the biological process category, the enriched terms were primarily associated with “astrocyte differentiation”, “glial cell development”, “cellular response to lipopolysaccharide”, and “glial cell differentiation”. The category of cellular component was mostly enriched in “interleukin 6-receptor complex”. The molecular functions category indicates notable associations with key processes involved in cell death and inflammation, such as “cysteine-type endopeptidase activity involved in execution phase of apoptosis”, “cysteine-type endopeptidase activity involved in apoptotic signaling pathway”, “potassium channel activator activity”, “interleukin-6 receptor binding”, “protein serine/threonine kinase inhibitor activity”, “tumor necrosis factor receptor superfamily binding”, and “cytokine receptor binding”. Furthermore, KEGG pathway enrichment analyses of six hub targets suggests potential links to biological pathways related to inflammation and neurological diseases, including “IL-17 signaling pathway”, “TNF signaling pathway”, “Toll-like receptor signaling pathway”, and “Alzheimer’s disease” (Figure 5).

### 2.5. Molecular Docking of AC-Derived Phytocompounds Against Key Hub Targets

**Figure 6:**
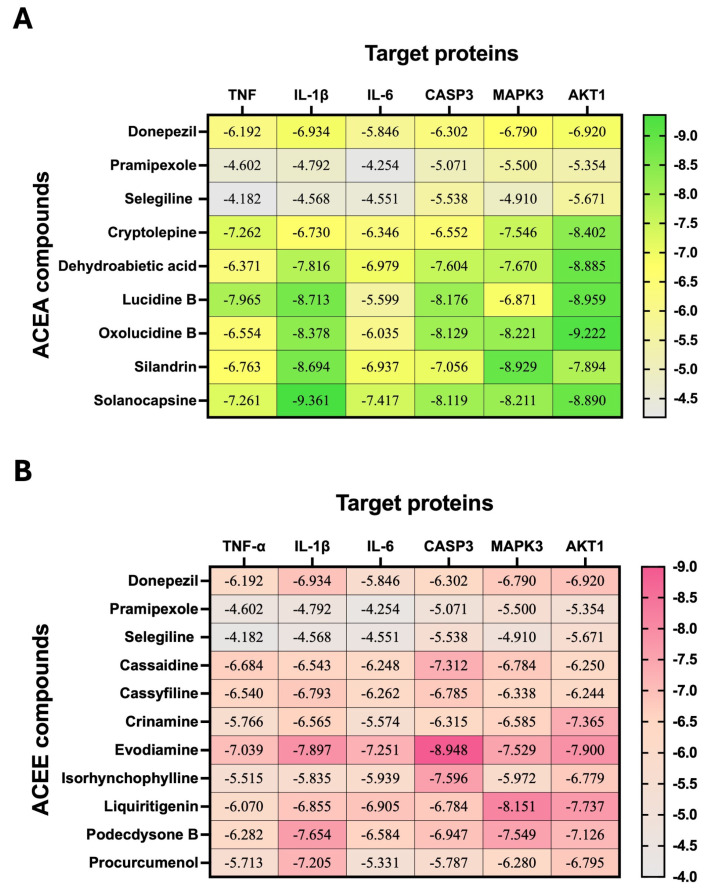
Molecular docking analysis of AC phytocompounds and hub targets. Heat maps representing the binding affinity scores (kcal/mol) of (**A**) the candidate compounds in ACEA and (**B**) ACEE, docked with six hub target proteins, including TNF-α, IL-1β, IL-6, CASP3, MAPK3, and AKT1.

**Figure 7:**
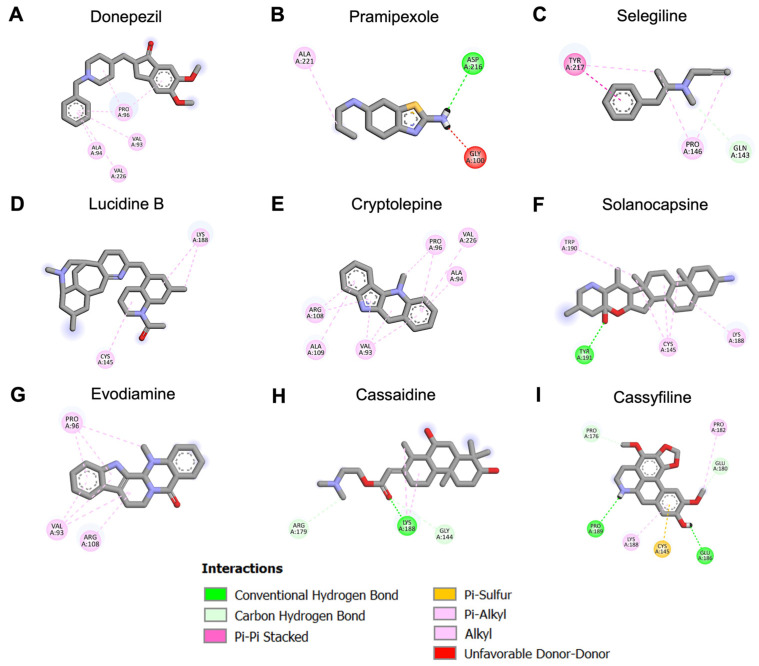
Schematic of amino acid residues and interaction types from molecular docking between TNF-α protein and reference drugs or AC phytocompounds. (**A**) Donepezil (AD drug), (**B**) Pramipexole (PD drug), (**C**) Selegiline (PD drug), (**D**) Lucidine B, (**E**) Cryptolepine, (**F**) Solanocapsine, (**G**) Evodiamine, (**H**) Cassaidine and (**I**) Cassyfiline.

**Figure 8:**
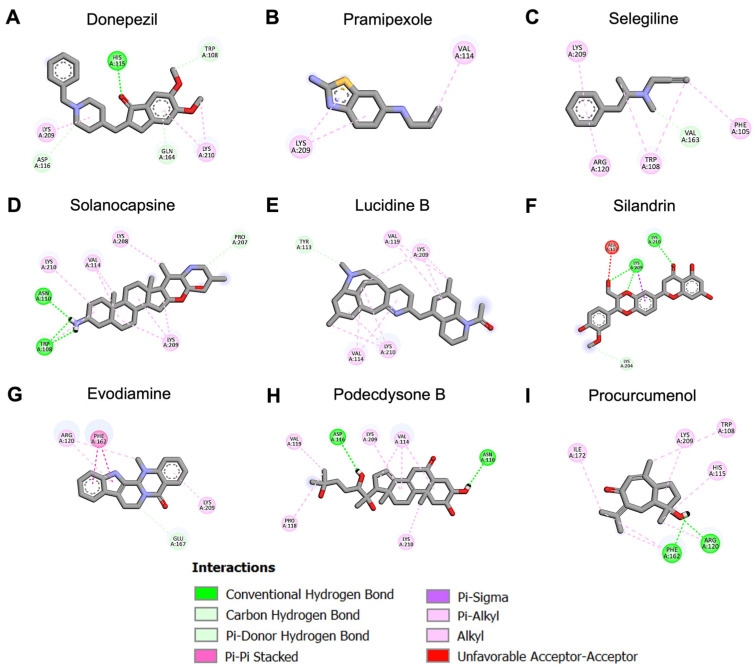
Schematic of amino acid residues and interaction types from molecular docking between IL-1β protein and reference drugs or AC phytocompounds. (**A**) Donepezil (AD drug), (**B**) Pramipexole (PD drug), (**C**) Selegiline (PD drug), (**D**) Solanocapsine, (**E**) Lucidine B, (**F**) Silandrin, (**G**) Evodiamine, (**H**) Podecdysone B, and (**I**) Procurcumenol.

**Figure 9:**
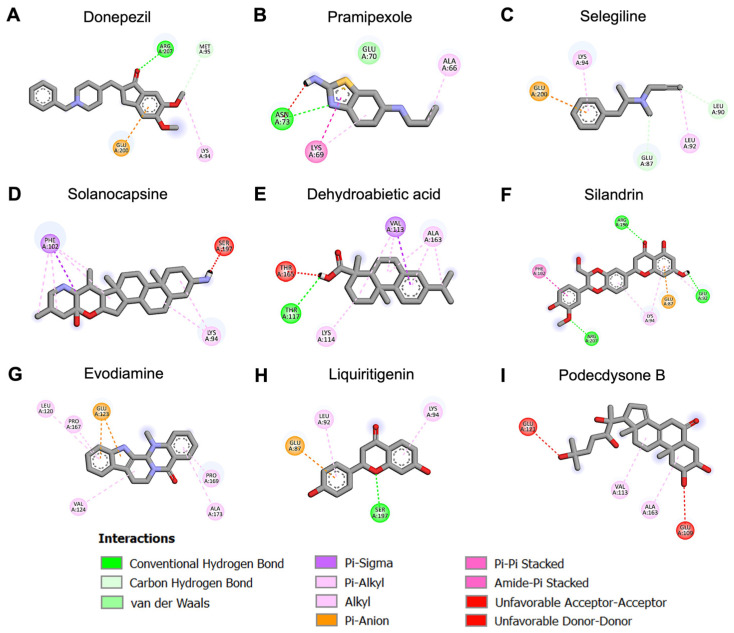
Schematic of amino acid residues and interaction types from molecular docking between IL-6 protein and reference drugs or AC phytocompounds. (**A**) Donepezil (AD drug), (**B**) Pramipexole (PD drug), (**C**) Selegiline (PD drug), (**D**) Solanocapsine, (**E**) Dehydroabietic acid, (**F**) Silandrin, (**G**) Evodiamine, (**H**) Liquiritigenin, and (**I**) Podecdysone B.

**Figure 10:**
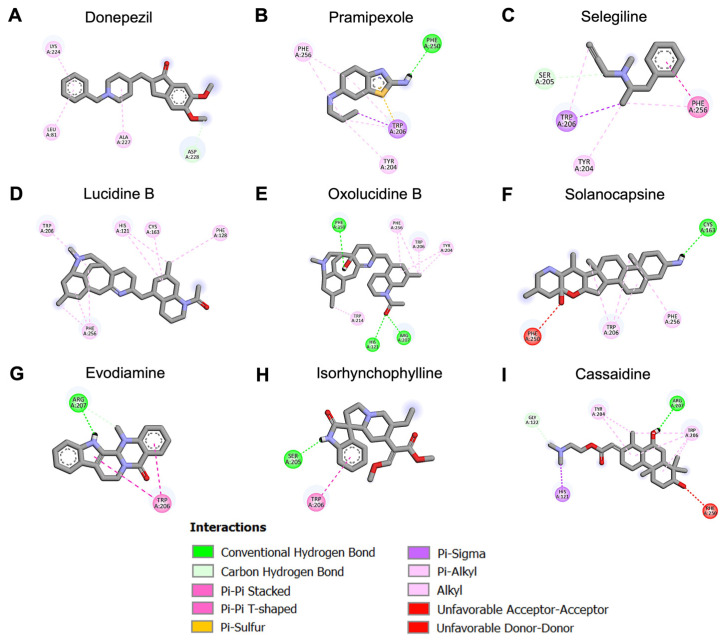
Schematic of amino acid residues and interaction types from molecular docking between CASP3 protein and reference drugs or AC phytocompounds. (**A**) Donepezil (AD drug), (**B**) Pramipexole (PD drug), (**C**) Selegiline (PD drug), (**D**) Lucidine B, (**E**) Oxolucidine B, (**F**) Solanocapsine, (**G**) Evodiamine, (**H**) Isorhynchophylline, and (**I**) Cassaidine.

**Figure 11:**
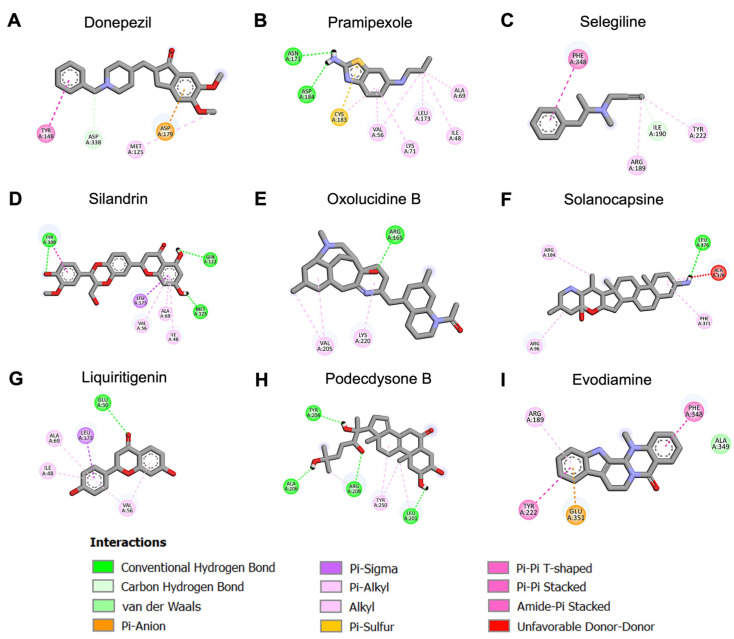
Schematic of amino acid residues and interaction types from molecular docking between MAPK3 protein and reference drugs or AC phytocompounds. (**A**) Donepezil (AD drug), (**B**) Pramipexole (PD drug), (**C**) Selegiline (PD drug), (**D**) Silandrin, (**E**) Oxolucidine B, (**F**) Solanocapsine, (**G**) Liquiritigenin, (**H**) Podecdysone B, and (**I**) Evodiamine.

**Figure 12:**
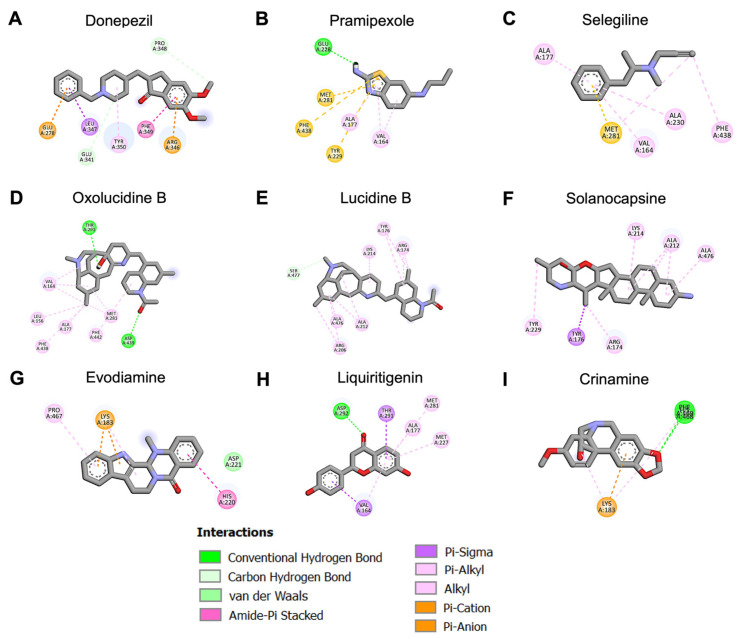
Schematic of amino acid residues and interaction types from molecular docking between AKT1 protein and reference drugs or AC phytocompounds. (**A**) Donepezil (AD drug), (**B**) Pramipexole (PD drug), (**C**) Selegiline (PD drug), (**D**) Oxolucidine B, (**E**) Lucidine B, (**F**) Solanocapsine, (**G**) Evodiamine, (**H**) Liquiritigenin, and (**I**) Crinamine.

Molecular docking was performed to validate the phytocompound–disease target interactions through assessing the binding affinities between AC phytocompounds and the identified six hub target proteins, including TNF-α (P01375), IL-1β (P01584), IL-6 (P05231), CASP3 (P42574), MAPK3 (P27361), and AKT1 (P31749). The FDA-approved drugs for AD (donepezil) and PD (pramipexole and selegiline) served as reference drugs in the analysis to compare their mechanistic actions with those of phytocompounds. The results of molecular docking analysis were summarised in Supplementary Tables S4–S15. A lower docking score indicates stronger binding interaction and greater stability between a ligand and protein target. The docking scores of the top three ranked AC phytocompounds for each target protein are demonstrated in Figure 6, along with their 2D protein-ligand interaction diagram illustrated in Figure 7, Figure 8, Figure 9, Figure 10, Figure 11 and Figure 12. As shown in Figure 6, the docking scores of the selected AC-derived phytocompounds across six hub proteins ranged from −5.599 to −9.361 kcal/mol (for ACEA) and −5.515 to −8.948 kcal/mol (for ACEE). These range values were higher than those of three reference drugs, which were in the range of −4.182 to −6.934 kcal/mol, indicating the stronger interactions of AC-derived phytocompounds with target proteins when compared to those of currently used AD and PD drugs. Additionally, the interactions of a known inhibitor of each target protein, including SPD-304 for TNF-α, Quercetin for IL-1β and IL-6, N-acetylcysteine for CASP3, Ulixertinib for MAPK3, and Ipatasertib for AKT1, were analyzed and served as the positive controls. The results are shown in Supplementary Tables S4–S15.

Among the ACEA-derived compounds (Figure 6A), lucidine B exhibited strongest binding affinity towards TNF-α and CASP3, while solanocapsine showed the most favourable docking energies against IL-1β and IL-6. Silandrin and oxolucidine B exhibited the highest binding affinity for MAPK3 and AKT1, respectively. Docking energies revealed that these compounds interacted with multiple amino acid residues within the active sites of the protein establishing binding interactions similar to those of reference drugs. For instance, solanocapsine interacted with residue TRP108 via hydrogen bonding in the IL-1 protein, as observed with donepezil. It also interacted with residues TRP108, LYS209, and LYS210 via hydrophobic interaction, as observed in both donepezil and selegiline (Figure 8). Oxolucidine B also formed hydrophobic interaction with residues MET281, VAL164, ALA177, and PHE438 of the AKT1 protein as observed with selegiline (Figure 12).

Among the ACEE identified compounds, evodiamine exhibited the most favourable binding affinities across the majority of the target proteins, including TNF-α, IL-1β, IL-6, CASP3, and AKT1. For MAPK3, liquiritigenin exhibited docking scores with evodiamine ranking third (Figure 6B). Further interaction analysis demonstrated that evodiamine could form hydrophobic interaction with residues TYR222, PHE348, and ARG189 on MAPK3 protein, as observed in selegiline. While liquiritigenin interacted with residues ALA69, ILE48, and VAL56 of MAPK3 via hydrophobic interactions, as found in pramipezole (Figure 11).

### 2.6. Effects of AC Extracts on the Expression of Hub Targets in LPS-Stimulated BV-2 Cells

**Figure 13:**
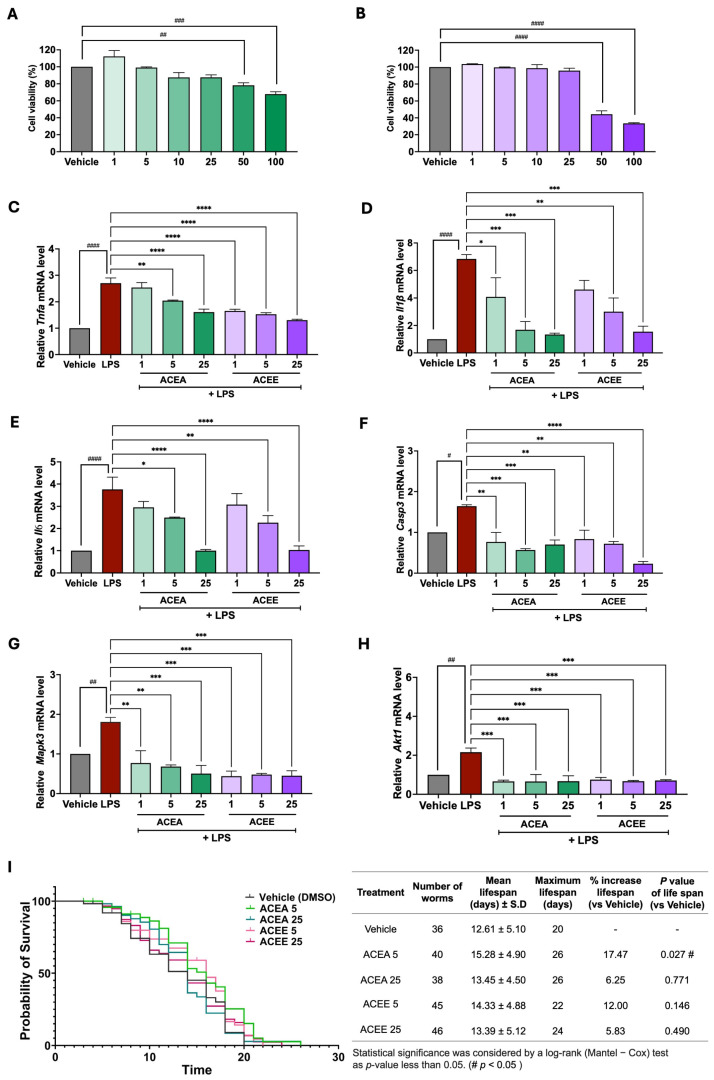
Experimental validation of potential therapeutic mechanisms of AC extracts in AD and PD predicted by network pharmacology analysis. Effects of (**A**) ACEA and (**B**) ACEE at varied concentrations from 1 to 100 µg/mL on BV-2 cell viability assessed by MTT assay. Effects of AC extracts at varied concentrations from 1 to 25 µg/mL in LPS-stimulated BV-2 cells on the mRNA expression of six hub targets, including (**C**) *Tnfa*, (**D**) *Il1b*, (**E**) *Il6*, (**F**) *Casp3*, (**G**) *Mapk3*, and (**H**) *Akt1*, assessed by qRT-PCR. Gene expression was normalised to *Actb* (β-actin) as an internal control and presented as fold change relative to the vehicle control. (**I**) Effects of AC extracts at 5 and 25 µg/mL on the lifespan of AD transgenic *C. elegans* strain CL2006. Data are represented as the mean ± SD from at least three independent experiments. A *p*-value < 0.05 was considered statistically significant, indicating a difference between groups. (#### *p* < 0.0001, ### *p* < 0.001, ## *p* < 0.01, # *p* < 0.05 vs. vehicle (DMSO control), **** *p* < 0.0001, *** *p* < 0.001, ** *p* < 0.01, * *p* < 0.05 vs. LPS treatment alone).

Neuroinflammation is recognised as a key driver of NDDs, particularly in the pathogenesis of AD and PD [19]. Therefore, further experiments using an in vitro cellular model of neuroinflammation were carried out to validate the network pharmacology prediction. Prior to assessing gene expression, the toxicity of AC extracts was evaluated using the MTT assay in BV-2 cells. The concentrations of extracts that exhibited more than 85% cell viability were considered non-cytotoxic and selected for subsequent experiments. The results showed that concentrations of 1–25 µg/mL of both ACEA and ACEE did not significantly reduce cell viability relative to vehicle control, indicating no cytotoxicity (Figure 13A,B). The non-toxic concentrations of 1, 5, and 25 µg/mL were selected to further investigate their effects on the gene expression of six hub targets (*Tnfa*, *Il1b*, *Il6*, *Casp3*, *Mapk3*, and *Akt1*) in LPS-stimulated BV-2 cells (a neuroinflammatory cell model). The RT-qPCR analysis demonstrated that LPS significantly increased mRNA expression of all hub targets in BV-2 microglial cells (*p* < 0.05) compared with vehicle control. However, treatment with AC extracts markedly alleviated LPS-induced changes in gene expression (*p* < 0.05) at almost all tested concentrations, compared with cells treated with LPS alone (Figure 13C–H).

### 2.7. Effects of AC Extracts on the Lifespan of C. elegans Model of AD

To further evaluate the anti-AD potential of AC extracts, a lifespan assay was conducted using the transgenic *C. elegans* CL2006 strain expressing Aβ, a hallmark protein in AD pathogenesis. The survival analysis demonstrated that ACEA at 5 μg/mL significantly promoted worm longevity, extending the mean lifespan by 17.47% compared with the vehicle control (Figure 13I). The mean lifespan of ACEE-treated worms was 12% higher, but not significant, than that of the control group. None of the extracts at 25 μg/mL significantly enhanced the worms’ lifespan.

## 3. Discussion

NDDs are projected to become increasingly crucial global health and medical challenges, largely due to demographic shifts towards ageing populations and the complex pathophysiology of these conditions, particularly AD and PD [20]. AD is characterised pathologically by the accumulation of insoluble Aβ aggregates and NFTs, which consist of precipitated or aggregated hyperphosphorylated tau protein. Clinically, the gradual onset of learning and memory dysfunction is a hallmark symptom of AD [21]. Conversely, PD is defined pathologically by the deterioration of dopaminergic neurons in the SN and by the presence of LBs and LNs, intracellular inclusions primarily composed of α-syn. The clinical presentation of PD encompasses both motor and non-motor symptoms. Motor manifestations commonly include bradykinesia, muscular rigidity, resting tremor, and disturbances of posture and gait [5]. Previous studies have shown that both diseases share overlapping pathogenesis and relevant biological features. For instance, α-syn has been implicated in aberrant synapse formation in the brains of AD patients and has been found in amyloid plaques in both human AD brain tissue and Tg2576 transgenic mouse models [22,23]. They share several fundamental pathological processes, including mitochondrial dysfunction, oxidative stress, and neuroinflammation [24]. Therefore, a deeper understanding of the molecular and cellular processes underlying AD and PD is essential for developing more effective therapeutic strategies and improving clinical outcomes. Current pharmacological treatments for these diseases primarily slow disease progression and are often associated with significant adverse effects. Consequently, there is growing interest in developing natural products and plant-derived compounds that provide therapeutic benefits with fewer side effects. In the present study, we employed multiple approaches, including untargeted metabolite profiling, network pharmacology, molecular docking, and experimental validation, to determine the neuroprotective properties of AC extracts (ACEA and ACEE) and to identify the relevant active phytocomponents.

The list of phytocompounds in AC extracts was evaluated for their drug-likeness and toxicity using Lipinski’s Rule of Five and toxicity risk prediction. A total of 42 compounds from ACEA and 17 compounds from ACEE met the criteria and exhibited non-toxicological properties. Among these components, quinic acid, myristoleic acid, ethyl nicotinate, and liquiritigenin have been previously reported in AC extracts [25,26]. The AD and PD targets were obtained from public databases, including GeneCards, COREMINE, and DisGeNET, whereas the phytocompound targets were derived from SwissTargetPrediction. Combining multiple databases can enhance data completeness and the reliability of disease target predictions, particularly in complex disorders. Subsequently, an intersection analysis of disease and phytocompound targets was performed, yielding 47 phytocompound–disease targets. These targets were subsequently determined by the PPI network, followed by an analysis of topological properties. The six hub targets including TNF-α, IL-1β, IL-6, CASP3, AKT1 and MAPK3 were selected based on their connectivity scores as top-ranking nodes across all topological algorithms. In terms of the biological processes associated with these targets, TNF-α, IL-1β, and IL-6 are well-recognised as key pro-inflammatory mediators involved in immune responses and play a crucial role in inflammatory-related diseases [27,28]. In AD, neuroinflammation is a pathological hallmark characterised by the presence of reactive microglia and astrocytes around Aβ plaques [29]. Excessive and dysregulated microglial activation triggered by Aβ and PD-related toxins contributes to AD and PD pathology by promoting the release of pro-inflammatory cytokines such as TNF-α, IL-1β, IL-6, and reactive oxygen species (ROS), triggering apoptotic pathways, and ultimately leading to neuronal degeneration [30,31,32]. Caspase 3, a key protein associated with neuronal apoptosis, has been reported to be increased in patients with AD and PD [33,34,35]. MAPK3 is involved in regulating cell proliferation, migration, and apoptosis. A previous study showed that knockdown of amyloid precursor protein (APP) suppressed the MAPK signaling pathway [36]. AKT, a serine/threonine kinase, is responsible for controlling cell survival, cell differentiation, and cell metabolism. Dysregulation of Akt1/mTOR signaling and its downstream effectors in the hippocampus contributes to impairments in memory function and a reduction in activity-dependent synaptic protein synthesis [37].

The identified hub targets were also subjected to GO and KEGG pathway enrichment analyses to elucidate the potential functional roles of AC phytocompounds in relation to AD and PD therapies. The results demonstrated that inflammatory responses were associated with the AC’s action, as evidenced by the three categories of GO terms related to glial cells, LPS, and pro-inflammatory cytokines, and by KEGG enrichment in TNF signaling and toll-like receptor signaling pathways. Glial cells (such as microglia and astrocytes) are immune cells in the central nervous system (CNS) that play crucial roles in brain homeostasis, immune responses, maintenance of the blood–brain barrier, and neuronal network function [38]. A previous study suggested that glial cells also play an important role in the clearance of Aβ and α-syn, indicating their relevance to AD and PD pathogenesis [39]. The development of both diseases has also been reported to be associated with endotoxin LPS signaling. This endotoxin could affect the homeostasis of brain Aβ by promoting its accumulation and deposition as plaques, as well as inducing tau pathology by enhancing the hyperphosphorylation of tau proteins [40]. Also, it induced cellular uptake and aggregation of α-syn, leading to the progression of PD [41]. Additionally, LPS is well known for eliciting robust inflammatory responses. Through binding to toll-like receptors, a family of pattern-recognition receptors, on brain immune cells, LPS can trigger excessive and dysregulated inflammation in the CNS, accompanied by oxidative stress and neuronal cell death, contributing to progressive loss of memory and behavioural deficits [42,43]. Notably, the KEGG analysis showed that all hub targets were enriched in AD-related pathways, highlighting the potential of AC for AD management.

The molecular docking study of AC phytocompounds against target proteins identified the top phytocompounds, which exhibited higher binding affinity than the reference drugss across all studied targets. This included four compounds from ACEA (dehydroabietic acid, oxolucidine B, silandrin, and solanocapsine) and two compounds from ACEE (evodiamine and podecdysone B). It is notable that amino acid interactions with the target proteins of AC phytocompounds were like those previously reported in other studies. For instance, liquiritigenin showed the higher binding affinity to MAPK3, interacting with LEU173, ALA69, ILE48, and VAL56 residues via a hydrophobic interaction with MAPK3, as observed with pramipezole (PD drug). This was consistent with previous studies showing that natural flavonoids and anthraquinones can interact with the active site of MAPK3 at several amino acid residues, including VAL56, ILE48, LEU173, ALA69, MET125, and CYS183 [44,45]. Oxolucidine B, which has the strongest binding affinity for MAPK3 and AKT1, could interact with MET281, VAL164, ALA177, and PHE438 residues through hydrophobic interactions with AKT1, as observed in selegiline (PD drug). This amino acid interaction was similar to that reported previously for siponimod, a drug for multiple sclerosis. It could interact with MET281, PHE161, ALA177, VAL164 and ARG4 residues through hydrophobic interactions with AKT1 [46]. Moreover, the docking results were consistent with previous experimental studies reporting that evodiamine significantly reduced the expression of TNF-α, IL-6, and IL-1β in BEAS-2B epithelial cells and AD mouse models [47,48]. Evodiamine also inhibited L-glutamate-induced apoptosis in the HT22 hippocampal cell line by reducing the expression of apoptotic markers, such as cleaved caspase-3 and Bcl-2-associated X protein (Bax) [49]. Liquiritigenin has been reported to exert anti-inflammatory effects in LPS-stimulated Raw264.7 macrophages by suppressing the expression of TNF-α, IL-1β, and IL-6 [50]. Additionally, it also suppressed cardiomyocytes’ apoptosis by attenuating the activation of TGF-β1/Smad2 and AKT/ERK signaling pathways [51].

Comparative analysis of the docking results revealed that several AC-derived phytocompounds exhibited lower binding energies when compared to those with positive controls. For instance, solanocapsine showed higher binding affinity against TNF-α, IL-1β, CASP3, MAPK3 and AKT1 compared to SPD-304, quercetin, N-acetylcysteine, ulixertinib and ipatasertib, respectively. Evodiamine and liquiritigenin also showed favourable binding energies than the positive controls for CASP3 and AKT1, respectively. Whereas these results, confirmed by comparisons with reference drugs, clearly indicate significant target engagement by AC phytocompounds, molecular docking studies are inadequate for a thorough understanding of the mechanism of action involved. It would be necessary to validate the results through additional methods, including molecular dynamics simulation, MM-GBSA/MM-PBSA binding free energy computation, and experimental approaches.

To validate the network pharmacology prediction, the effects of AC extracts on the gene expression of six hub targets were further investigated in BV-2 microglial cells stimulated with LPS to induce inflammatory responses. Previous studies have shown that LPS could enhance neuroinflammatory features in AD and PD models [52,53]. In this present study, LPS significantly increased mRNA levels of all target genes, which were markedly suppressed by ACEA and ACEE treatments. This is consistent with our previous report that ACEE could lower the expressions of *Tnfa*, *Il1b*, and *Il6* in anthracene-induced BV-2 cells [16]. The expression of these pro-inflammatory mediators was reduced in a study of human T lymphocyte cells (Jurkat) in response to aqueous and chloroform extracts of AC [26]. Additionally, this study showed that both ACEA and ACEE could prolong the lifespan of the transgenic *C. elegans* AD model, supporting the potential of AC as an AD therapeutic. Nevertheless, this enhancing effect was not significant at higher concentrations of both extracts. This might have occurred due to an inappropriate dose of the extract, and a previous report noted that increasing the dose could reduce drug effect [54]. However, there are diverse toxicological effects and risks of AC that should be considered. Previous research mentioned the effects of AC on oral submucosal fibrosis, which can develop into oral cancer [55]. Especially, arecoline, a major component in AC, has been reported to have an effect on enhancing the fibrosis development and mediating oral carcinogenicity risks [56]. It should be noted that while AC and its derived phytocompounds possess several beneficial pharmacological properties, there are serious toxicological issues, especially carcinogenicity, that must be of concern in practical use.

Nevertheless, several limitations of this study should also be considered. There are relatively few target proteins used for GO and KEGG enrichment analyses. Therefore, the information provided on the biological functions of the proteins and their involvement in specific metabolic pathways cannot be taken as conclusive, as it represents a mechanistic hypothesis about what is happening with the proteins in question rather than a definitive biological function annotation. Increasing the size of the target protein pool by expanding the selection criteria is crucial for future studies. In future research, all targets and their associated mechanisms should be studied at the protein level (e.g., Western blotting, immunostaining, enzyme-linked immunosorbent assay or ELISA) to confirm their functional involvement and provide a more comprehensive understanding of the signaling pathways underlying the therapeutic potential of AC extracts. Considering that the therapeutic efficacy of the extract often relies on the synergistic interactions and combined effects of its phytochemical composition, rather than isolated compounds [57,58], the single AC-derived phytocompounds and their combinations should be experimentally validated to identify the complex biological interactions in cell culture models of neurodegeneration, as limited by computational studies. Such cellular models and synergism quantification approaches include glutamate-treated HT-22 hippocampal neuronal cells for AD study [59], 1-Methyl-4-phenylpyridinium (MPP^+^)-treated SH-SY5Y neuronal cells for PD study [60], LPS-stimulated BV-2 cell death for neurodegeneration study [61], the Chou-Talalay combination index method, and the Mixlow method [62,63]. Moreover, experiments using animal models and human trials are crucial in comprehensively evaluating both the safety and efficacy of AC for clinical applications.

## 4. Materials and Methods

### 4.1. Chemicals and Reagents

Lipopolysaccharides (LPS) from *E. coli* O55:B5 and Dulbecco’s Modified Eagle’s Medium (DMEM) were obtained from Sigma-Aldrich (St. Louis, MO, USA). Fetal bovine serum (FBS) and 0.25% trypsin-EDTA were purchased from Gibco BRL (Life Technologies, Paisley, UK). 3-(4,5-dimethylthiazol-2-yl)-2,5-diphenyltetrazolium bromide (MTT) was purchased from Bio Basic (Markham, ON, Canada). GENEzol reagent was obtained from Invitrogen (Carlsbad, CA, USA). AccuPower RT-premix and all primers were obtained from Bioneer (Daejeon, Republic of Korea). The iTaq™ Universal SYBR^®^ Green Supermix was purchased from BioRad (Hercules, CA, USA). Dimethyl sulfoxide (DMSO) and analytical-grade solvents were purchased from RCI Labscan (Bangkok, Thailand).

### 4.2. Preparation of AC Extracts

The fruits of AC were collected from a local garden in Surat Thani province, Thailand, and botanically authenticated by the herbarium of Kasin Suvatabhandhu, Department of Botany, Faculty of Science, Chulalongkorn University. The voucher specimen was deposited at the herbarium (BCU 016434). Prior to extraction, the fruits were first separated from the husks, cleaned, dried, and ground into a fine powder. Approximately 40 g of the powder was extracted with 400 mL of ethyl acetate or absolute ethanol in a Soxhlet apparatus. The obtained extracts were then concentrated by rotary evaporation. Each crude extract was dissolved in DMSO to prepare a stock solution at 100 mg/mL and stored at −20 °C in the dark before use. Ethyl acetate and ethanolic extracts of AC were designated as ACEA and ACEE, respectively.

### 4.3. Phytochemical Profiling Analysis

Liquid chromatography (LC) coupled with a quadrupole time-of-flight (QTOF) mass spectrometry (MS) (Bruker Daltonics, Bremen, Germany) was utilised to explore the phytochemical profiling in ACEA and ACEE. A mobile phase used in LC consists of (A) 2.5 mM ammonium formate buffer in water and (B) 2.5 mM ammonium formate buffer in methanol. A 2 µL of extract sample was injected into an Intensity Solo C18 column with an inner diameter of 2.1 mm, a particle size of 1.8 µm, and a length of 100 mm. The column temperature was maintained at 40 °C. A flow rate was adjusted to 0.4 mL/min with a gradient containing 0.1% B for 0–10 min, 25% B for 10–12 min, 80% B for 12–21 min, 90% B for 21–23 min, and 0.1% B for 23–26 min. The MS/MS analysis was acquired with 500 volts of electrospray ionisation (ESI) in the positive ion mode, which was applied as a detector. The compounds were tentatively identified based on mass-to-charge ratio (*m*/*z*) values and MS spectra using MetFrag software version 2.6.11 (https://msbi.ipb-halle.de/MetFrag/, accessed on 28 April 2025) and the KNApSAcK database version 1.200.03 (https://www.knapsackfamily.com/KNApSAcK/, accessed on 28 April 2025) within a mass tolerance of 30 parts-per-million (ppm).

### 4.4. Network Pharmacology Analysis

#### 4.4.1. Prediction of Phytocompound Targets

The potential protein targets of the phytocompounds observed in AC extracts were identified using SwissTarget Prediction (http://www.swisstargetprediction.ch/, accessed on 9 May 2025). This database facilitates target prediction by integrating structural features of compounds with experimentally derived binding affinity data across diverse proteins. The compounds’ canonical SMILES strings were entered into the database to obtain their predicted target proteins.

#### 4.4.2. Drug-Likeness and Toxicological Evaluation of Phytocompounds

The drug-likeness and oral bioavailability of the AC phytocompounds were evaluated using the criteria of Lipinski’s Rule of Five, a popular guideline used in drug discovery. The drug-likeness of the compounds was considered based on key physicochemical properties, including molecular weight (≤500 Da), hydrogen bond donors (≤5), hydrogen bond acceptors (≤10), and high lipophilicity expressed as the logarithm of the partition coefficient (logP ≤ 5). Additionally, toxicity risk prediction of phytocompounds was conducted to assess four types of toxicity: mutagenic, tumorigenic, reproductive, and irritant.

#### 4.4.3. Identification of AD and PD-Related Targets

The targets associated with AD and PD were identified using the keywords “Alzheimer’s” and “Parkinson’s” and searched across three publicly available databases, including GeneCards [64], COREMINE [65], and DisGeNET [64]. The datasets of disease-related targets retrieved from all databases were merged and subjected to overlap analysis to identify shared common targets among the three datasets and subsequently between the two different diseases.

#### 4.4.4. Identification of Overlapping Targets Between the Phytocompounds and Diseases

The datasets of phytocompound targets and AD- and PD-related targets were integrated and subjected to Venn diagram analysis using Venny 2.1 (https://bioinfogp.cnb.csic.es/tools/venny/, accessed on 19 May 2025) to identify overlapping targets between the phytocompound- and disease-related targets [66].

#### 4.4.5. Construction of Protein–Protein Interaction Networks and Topology Analysis

The intersected targets between phytocompounds and diseases were imported into the STRING database (https://string-db.org/, accessed on 11 June 2025) to predict and generate protein–protein interaction (PPI) networks, with “*Homo sapiens*” as the reference organism. STRING is a comprehensive database that collects and integrates information on protein interactions, both physical and functional associations, from multiple sources, including prior studies, experimental assays, and computational predictions. To construct the PPI, predicted associations were filtered using a medium confidence threshold (≥0.4). The criterion chosen was aimed at balancing sensitivity and specificity optimally to ensure that only interactions supported by enough evidence would remain for further functional analysis [67,68]. Then, the resulting interaction network was transferred and displayed in Cytoscape 3.10.0 (https://www.cytoscape.org/, accessed on 16 June 2025). Next, the topological analysis of the constructed PPI networks was applied to evaluate the connectivity profiles of targets (represented as nodes in the network) and to identify highly connected nodes based on their degree (number of interactions) using the Cytoscape plugin CytoHubba. Topological analysis algorithms determined in this study include closeness centrality, degree centrality, and edge percolation centrality (EPC) [18,69]. The top-ranking nodes (hubs) under each algorithm were selected for further analysis.

#### 4.4.6. Functional Enrichment and Pathway Analysis

To evaluate the functional roles of identified target genes, including biological process, molecular function, and cellular component, Gene Ontology (GO) and Kyoto Encyclopedia of Genes and Genomes (KEGG) pathway enrichment analyses were conducted using the ShinyGo 0.85 online version. For the functional enrichment analysis, the background network was defined as the entire genome. This setup provided a comprehensive list of enriched pathways, along with their associated genes, the number of background genes, *p*-values, and false discovery rate (FDR; Q) values.

### 4.5. Molecular Docking

Molecular docking of the candidate ligands against the target proteins was performed using AutoDock Vina (Version 1.2.3) on a Linux platform, employing a script-based automated workflow for multiple ligand processing. The predicted 3D structures of target proteins were retrieved from the AlphaFold Protein Structure Database using their UniProt accession numbers (UniProt IDs) [70]. Prior to docking, the target protein structure was processed and subjected to energy minimization in UCSF Chimera 1.19 employing the AMBER ff14SB force field to obtain a stable conformation. Further, protein preparation was performed by adding all nonpolar hydrogens and assigning Gasteiger charges according to the AutoDock Vina requirements [71,72]. All ligands and protein structures were converted to the PDBQT format using AutoDockTools (ADT) 1.5.7. Moreover, the structures of the compounds were geometry optimised and energy minimization of the ligands were done using the MMFF94 force field with 1000 minimization steps (*n* = 1000) [73]. Docking was performed with default exhaustiveness parameters unless otherwise specified. In addition, we have employed a blind docking technique. This method allowed for the objective identification of possible ligand-binding sites throughout the protein surface by defining the grid box to include the full protein structure [74]. For every docking simulation, grid box dimensions and center coordinates were set to encompass the entire protein shape. The docking conformation with the lowest binding energy was selected for further analysis. Protein–ligand interactions, including hydrogen bonds, hydrophobic contacts, and π-interactions, were visualised and analyzed using BIOVIA Discovery Studio 2020 (San Diego, CA, USA) [75].

### 4.6. In Vitro Experimental Validation

#### 4.6.1. Cell Culture

The immortalised BV-2 mouse microglial cell line (Cat. #ABC-TC212S) was obtained from Dr. Monruedee Sukprasansap (Institute of Nutrition, Mahidol University). BV-2 cells were cultured in DMEM supplemented with 10% FBS, 100 U/mL penicillin, and 100 µg/mL streptomycin in a humidified incubator containing 5% CO_2_ at 37 °C. Cells were cultured until approximately 80% confluence before initiating the experiments. BV-2 cells were stimulated with 1 μg/mL LPS to establish the neuroinflammatory model and further incubated in the presence or absence of AC extracts for the specified time periods.

#### 4.6.2. Cell Viability

MTT assay was conducted to assess the cytotoxicity of AC extracts on BV-2 cells. Briefly, cells were seeded in a 96-well plate overnight, then exposed to various doses of AC extracts for 24 h. After completing the incubation period, MTT solution (5 mg/mL) was added to the wells and incubated for another 4 h. Finally, the purple formazan products were dissolved in DMSO, and the absorbance was read at 570 nm using an EnSpire^®^ Multimode Plate Reader (PerkinElmer, Waltham, MA, USA). Data are presented as percentages relative to control cells (untreated cells).

#### 4.6.3. RNA Extraction and RT-qPCR Analysis

Quantitative reverse transcription polymerase chain reaction (RT-qPCR) was performed to determine the expression of hub targets following exposure to AC extracts, validating the results of network pharmacology analysis. After stimulating BV-2 cells with LPS, with or without AC extracts, for 3 h (for *Tnfa*, *Il1b*, *Il6*) or 24 h (for *Casp3*, *Mapk3*, *Akt1*), total RNA was extracted using GENEzol reagent (Invitrogen) according to the manufacturer’s protocol. Then, 1 µg/mL of RNA was reverse transcribed into complementary DNA using AccuPower RT-premix (Bioneer), which was then used as a template for amplification using iTaq Universal SYBR Green Supermix (BioRad) under the following conditions: 1 cycle of 95 °C for 10 min, followed by 45 cycles of 95 °C for 20 s and 58 °C for 40 s, in a CFX Opus Real-Time PCR System (BioRad). Relative gene expression was calculated using the 2^−ΔΔCt^ method with *Actb* (β-actin) as the internal control. All primer sequences used in this analysis are listed in Supplementary Table S16.

### 4.7. In Vivo Experimental Validation

#### 4.7.1. *C. elegans* Maintenance and Synchronization

The transgenic *C. elegans* strain expressing Aβ (CL2006) used in this study was obtained from Caenorhabditis Genetics Center (CGC). The worms were maintained on nematode growth medium (NGM) agar seeded with *Escherichia coli* OP50 at 15 °C. Age-synchronised worms were obtained by bleaching with a hypochlorite–sodium hydroxide solution to reach the first larval stage (L1) and subsequently incubated on a fresh NGM agar plate until the fourth larval stage (L4).

#### 4.7.2. *C. elegans* Lifespan Assay

The lifespan assay of the transgenic *C. elegans* CL2006 strain was carried out at 20 °C to induce Aβ expression and toxicity in the worms. The age-synchronised L4 worms were placed on fresh NGM plates containing *E. coli* OP50 with AC extracts at 5 or 25 μg/mL, or vehicle (DMSO control), and the plates were exchanged every 2 days to prevent overcrowding and starvation. The worms were monitored daily until all animals died, and their status was recorded as alive or dead when they failed to respond to gentle prodding with a platinum wire.

### 4.8. Statistical Analysis

The cell-based experiments were performed in triplicate and the results were presented as the mean ± standard deviation (SD). One-way ANOVA followed by Tukey’s multiple comparison test was performed to compare the mean difference among groups. Statistical significance was considered when the *p*-value was less than 0.05. For *C. elegans* lifespan assay, the log-rank (Mantel–Cox) test was used to compare survival curves between groups.

## 5. Conclusions

The present study employed multiple approaches, including network pharmacology, molecular docking, and in vitro and in vivo experiments, to elucidate the potential of AC extracts alongside the underlying mechanisms of action in targeting neurodegenerative diseases, particularly AD and PD. Six hub targets involved in the pathways related to neuroinflammation, apoptosis, and AD, including TNF-α, IL-1β, IL-6, CASP3, MAPK3, and AKT1, were identified in network pharmacology as potential targets of both diseases, and their expression levels were upregulated in the LPS-stimulated BV-2 neuroinflammation model. Based on the docking analysis and RT-qPCR validation, it was shown that AC exerted its neuroprotective activities through the downregulation of gene expression. Lucidine B, oxolucidine B, solanocapsine, evodiamine, and liquiritigenin are the principal active components of AC that contribute to the effects. In addition, AC extracts demonstrated lifespan-extending properties in a transgenic *C. elegans* AD model. However, further experimental validation is warranted to strengthen our findings and confirm the proposed mechanism of action of AC extracts and AC-derived compounds. Collectively, these findings have provided a greater understanding of how AC acts on multiple targets in treating AD and PD, which could help in developing therapeutic approaches to both neurodegenerative diseases in the future.

## Figures and Tables

**Table 1:** Lipinski’s rule of five and the toxicological properties of phytocompounds in ACEA.

NO	Name	Molecular Weight	cLogP	H- Acceptors	H- Donors	Mutagenic	Tumorigenic	Reproductive Effective	Irritant
1	(2S)-Flavanone	224.258	3.1928	2	0	none	none	none	none
2	1,2,3,4-Tetrahydro-Beta-Carboline-3-Carboxylic Acid	216.239	−1.7962	4	3	none	none	none	none
3	4-Methylthio-2-oxobutanoic acid	148.182	−0.2941	3	1	none	none	none	none
4	Aspidinol	224.255	2.0221	4	2	none	none	none	none
5	Buddledin A ^$^	276.375	3.8959	3	0	none	none	none	none
6	Carapanaubine	428.483	0.7022	8	1	none	none	none	none
7	Cimifugin	306.313	1.4452	6	2	none	none	none	none
8	Cinchonamine	296.413	3.1103	3	2	none	none	none	none
9	Conessine	356.596	4.013	2	0	none	none	none	none
10	Cryptolepine	232.285	2.3465	2	0	none	none	none	none
11	Dehydroabietic acid	300.44	4.439	2	1	none	none	none	none
12	Dehydrocurdione	234.338	4.4255	2	0	none	none	none	none
13	Dihydroshikonofuran	260.332	3.7074	3	2	none	none	none	none
14	Dimethamine	408.544	1.453	6	0	none	none	none	none
15	Eburnamonine	294.397	4.042	3	0	none	none	none	none
16	Ecgonone methyl ester	197.233	0.0661	4	0	none	none	none	none
17	Erythrocentaurin	176.171	1.3726	3	0	none	none	none	none
18	(+)-Eudesmin ^$^	386.442	2.7218	6	0	none	none	none	none
19	Flindersine	227.262	1.756	3	1	none	none	none	none
20	Homostachydrine	157.212	−5.1309	2	0	none	none	none	none
21	Kaurenoic Acid	302.456	4.1175	2	1	none	none	none	none
22	L-Glutamine	146.145	−3.7564	5	3	none	none	none	none
23	Lathyrine	182.182	−3.3662	6	3	none	none	none	none
24	Methylitaconate	144.126	−0.0274	4	2	none	none	none	none
25	Montanol ^$^	352.513	4.0777	4	2	none	none	none	none
26	N-Ethylacetamide	87.1215	0.0609	2	1	none	none	none	none
27	Oxolucidine B	483.738	4.1223	5	1	none	none	none	none
28	Phellodendrine	342.414	−0.7916	4	2	none	none	none	none
29	Phytosphingosine	317.512	3.8577	4	4	none	none	none	none
30	Podecdysone B ^$^	462.624	2.9476	6	5	none	none	none	none
31	Quinic acid ^$^	192.166	−2.3347	6	5	none	none	none	none
32	Sebacic acid	202.249	2.0598	4	2	none	none	none	none
33	Senkyunolide B	204.224	2.7572	3	1	none	none	none	none
34	Lucidine B	467.739	4.9457	4	0	none	none	none	none
35	Silandrin	466.441	2.9787	9	4	none	none	none	none
36	Solanocapsine	430.674	3.5925	4	3	none	none	none	none
37	Sphinganine	301.513	4.8795	3	3	none	none	none	none
38	Taxodione	314.423	3.555	3	1	none	none	none	none
39	Tetryl	287.144	−2.2839	9	0	none	none	none	none
40	Thiarubrine A	228.339	4.7119	0	0	none	none	none	none
41	Tremetone	202.252	2.9566	2	0	none	none	none	none
42	Vasconine	266.319	−0.0224	2	0	none	none	none	none

**^$^** Phytocompounds found in both ACEA and ACEE.

**Table 2:** Lipinski’s rule of five and the toxicological properties of phytocompounds in ACEE.

NO	Name	Molecular weight	cLogP	H- Acceptors	H- Donors	Mutagenic	Tumorigenic	Reproductive Effective	Irritant
1	Buddledin A ^$^	276.375	3.8959	3	0	none	none	none	none
2	Cassaidine	407.593	2.8904	5	2	none	none	none	none
3	Cassyfiline	341.362	2.9924	6	2	none	none	none	none
4	Crinamine	301.341	0.9675	5	1	none	none	none	none
5	Ethyl nicotinate	151.164	0.978	3	0	none	none	none	none
6	(+)-Eudesmin ^$^	386.442	2.7218	6	0	none	none	none	none
7	Evodiamine	303.364	2.7404	4	1	none	none	none	none
8	Isorhynchophylline	384.474	1.888	6	1	none	none	none	none
9	Liquiritigenin	256.256	2.5014	4	2	none	none	none	none
10	Montanol ^$^	352.513	4.0777	4	2	none	none	none	none
11	Myristoleic acid	226.358	4.9015	2	1	none	none	none	none
12	Pithecolobine	382.634	3.6501	5	4	none	none	none	none
13	Podecdysone B ^$^	462.624	2.9476	6	5	none	none	none	none
14	Procurcumenol	234.338	3.1209	2	1	none	none	none	none
15	Quinic acid ^$^	192.166	−2.3347	6	5	none	none	none	none
16	Stachydrine	143.185	−5.4729	2	0	none	none	none	none
17	Trigonelline	137.138	−5.8558	2	0	none	none	none	none

**^$^** Phytocompounds found in both ACEA and ACEE.

## Data Availability

The data generated and/or analysed during this study are included in this published article and its Supplementary Materials file.

## Supplementary Materials

The following supporting information can be downloaded at: https://www.mdpi.com/article/10.3390/ijms27125169/s1.

## Abbreviations

The following abbreviations are used in this manuscript:

Aβ	Amyloid-beta
ACEA	Ethyl acetate extract of AC
ACEE	Ethanolic extract of AC
AC	*Areca catechu* L.
AD	Alzheimer’s disease
AKT1	AKT serine/threonine kinase 1
CASP3	Caspase 3
*C. elegans*	*Caenorhabditis elegans*
DMEM	Dulbecco’s Modified Eagle’s Medium
DMSO	Dimethyl sulfoxide
EPC	Edge percolation centrality
FBS	fetal bovine serum
FDR	false discovery rate
GO	Gene Ontology
Hba	Hydrogen bond acceptor
Hbd	Hydrogen bond donor;
IL-1β	Interleukin-1 beta
IL-6	Interleukin-6
KEGG	Kyoto Encyclopedia of Genes and Genomes
LBs	Lewy bodies
LC	Liquid chromatography
LNs	Lewy neurites
LogP	Logarithm of the partition coefficient
LPS	Lipopolysaccharides
MAPK3	Mitogen-activated protein kinase 3
MS	Mass spectrometry
MTT	3-(4,5-dimethylthiazol-2-yl)-2,5-diphenyltetrazolium bromide
MW	Molecular weight
NDDs	Neurodegenerative disorders
NFTs	Neurofibrillary tangles
NGM	Nematode growth medium
PD	Parkinson’s disease
PPI	Protein-protein interaction
QTOF	Quadrupole time-of-flight
RT-qPCR	Quantitative reverse transcription polymerase chain reaction
SN	Substantia nigra
α-syn	α-synuclein
TNF-α	Tumor necrosis factor-alpha
UniProt ID	UniProt accession number

## References

[1] Gadhave D.G., Sugandhi V.V., Jha S.K., Nangare S.N., Gupta G., Singh S.K., Dua K., Cho H., Hansbro P.M., Paudel K.R. (2024). Neurodegenerative disorders: Mechanisms of degeneration and therapeutic approaches with their clinical relevance. Ageing Res. Rev..

[2] Tiwari S., Atluri V., Kaushik A., Yndart A., Nair M. (2019). Alzheimer’s disease: Pathogenesis, diagnostics, and therapeutics. Int. J. Nanomed..

[3] Stocchi F., Bravi D., Emmi A., Antonini A. (2024). Parkinson disease therapy: Current strategies and future research priorities. Nat. Rev. Neurol..

[4] Zheng Q., Wang X. (2025). Alzheimer’s disease: Insights into pathology, molecular mechanisms, and therapy. Protein Cell.

[5] Poewe W., Seppi K., Tanner C.M., Halliday G.M., Brundin P., Volkmann J., Schrag A.-E., Lang A.E. (2017). Parkinson disease. Nat. Rev. Dis. Prim..

[6] Sequeira L., Benfeito S., Fernandes C., Lima I., Peixoto J., Alves C., Machado C.S., Gaspar A., Borges F., Chavarria D. (2024). Drug Development for Alzheimer’s and Parkinson’s Disease: Where Do We Go Now?. Pharmaceutics.

[7] Kirubakaran D. (2025). Herbal remedies for Alzheimer’s disease: Neuroprotective mechanisms and cognitive enhancement potential. Digit. Chin. Med..

[8] Tong T., Xu A., Tan S., Jiang H., Liu L., Deng S., Wang H. (2024). Biological Effects and Biomedical Applications of Areca Nut and Its Extract. Pharmaceuticals.

[9] Salehi B., Konovalov D.A., Fru P., Kapewangolo P., Peron G., Ksenija M.S., Cardoso S.M., Pereira O.R., Nigam M., Nicola S. (2020). *Areca catechu*—From farm to food and biomedical applications. Phytother. Res..

[10] Amudhan M.S., Vava Mohideen H., Hebbar K. (2012). A review on phytochemical and pharmacological potential of *Areca catechu* L. Seed. Int. J. Pharm. Sci. Res..

[11] Sun Y., Feng J., Hou W., Qi H., Liu Y. (2024). Comprehensive insights into areca nut: Active components and omics technologies for bioactivity evaluation and quality control. Front. Pharmacol..

[12] Yi S., Zou L., Li Z., Sakao K., Wang Y., Hou D.X. (2022). In Vitro Antioxidant Activity of Areca Nut Polyphenol Extracts on RAW264.7 Cells. Foods.

[13] Bozorgi M., Najafi Z., Omidpanah S., Sadri A., Narimani Z., Homayouni Moghadam F., Edraki N., Akbarzadeh T., Saeedi M. (2021). Investigation of anti-Alzheimer’s activity of aqueous extract of areca nuts (*Areca catechu* L.): In vitro and in vivo studies. Bol. Latinoam. Caribe Plantas Med. Aromat..

[14] Pradeep S., Prabhuswaminath S.C., Reddy P., Srinivasa S.M., Shati A.A., Alfaifi M.Y., Eldin I.E.S., Achar R.R., Silina E., Stupin V. (2022). Anticholinesterase activity of *Areca catechu*: In Vitro and in silico green synthesis approach in search for therapeutic agents against Alzheimer’s disease. Front. Pharmacol..

[15] Xu Z., Adilijiang A., Wang W., You P., Lin D., Li X., He J. (2019). Arecoline attenuates memory impairment and demyelination in a cuprizone-induced mouse model of schizophrenia. Neuroreport.

[16] Janpaijit S., Sukprasansap M., Tencomnao T., Prasansuklab A. (2024). Anti-Neuroinflammatory Potential of Areca Nut Extract and Its Bioactive Compounds in Anthracene-Induced BV-2 Microglial Cell Activation. Nutrients.

[17] Noor F., Tahir Ul Qamar M., Ashfaq U.A., Albutti A., Alwashmi A.S.S., Aljasir M.A. (2022). Network Pharmacology Approach for Medicinal Plants: Review and Assessment. Pharmaceuticals.

[18] Chhotaray S., Verma K., Sahoo P., Jal S. (2025). Network pharmacology and molecular docking analysis for elucidation of mechanism of action and molecular targets of *Commiphora wightii* in treatment of atherosclerosis. Advancement in Animal Handling and Generative AI for Pre-clinical Studies.

[19] Tylutka A., Żabiński P., Walas Ł., Zembron-Lacny A. (2024). Neuroinflammation as a Link in Parkinson’s and Alzheimer’s Diseases: A Systematic Review and Meta-Analysis. Aging Dis..

[20] Hou Y., Dan X., Babbar M., Wei Y., Hasselbalch S.G., Croteau D.L., Bohr V.A. (2019). Ageing as a risk factor for neurodegenerative disease. Nat. Rev. Neurol..

[21] Kamatham P.T., Shukla R., Khatri D.K., Vora L.K. (2024). Pathogenesis, diagnostics, and therapeutics for Alzheimer’s disease: Breaking the memory barrier. Ageing Res. Rev..

[22] Kim S., Seo J.H., Suh Y.H. (2004). Alpha-synuclein, Parkinson’s disease, and Alzheimer’s disease. Park. Relat. Disord..

[23] Yang F., Uéda K., Chen P., Ashe K.H., Cole G.M. (2000). Plaque-associated alpha-synuclein (NACP) pathology in aged transgenic mice expressing amyloid precursor protein. Brain Res..

[24] Xie A., Gao J., Xu L., Meng D. (2014). Shared mechanisms of neurodegeneration in Alzheimer’s disease and Parkinson’s disease. BioMed Res. Int..

[25] Liu P.-F., Chang Y.-F. (2023). The Controversial Roles of Areca Nut: Medicine or Toxin?. Int. J. Mol. Sci..

[26] Faouzi M., Neupane R.P., Yang J., Williams P., Penner R. (2018). Areca nut extracts mobilize calcium and release pro-inflammatory cytokines from various immune cells. Sci. Rep..

[27] Al-Qahtani A.A., Alhamlan F.S., Al-Qahtani A.A. (2024). Pro-Inflammatory and Anti-Inflammatory Interleukins in Infectious Diseases: A Comprehensive Review. Trop. Med. Infect. Dis..

[28] Kany S., Vollrath J.T., Relja B. (2019). Cytokines in Inflammatory Disease. Int. J. Mol. Sci..

[29] Edler M.K., Munger E.L., Maycon H., Hopkins W.D., Hof P.R., Sherwood C.C., Raghanti M.A. (2023). The association of astrogliosis and microglial activation with aging and Alzheimer’s disease pathology in the chimpanzee brain. J. Neurosci. Res..

[30] Ren M., Han M., Wei X., Guo Y., Shi H., Zhang X., Perez R.G., Lou H. (2017). FTY720 Attenuates 6-OHDA-Associated Dopaminergic Degeneration in Cellular and Mouse Parkinsonian Models. Neurochem. Res..

[31] Zhu Y., Guo X., Li S., Wu Y., Zhu F., Qin C., Zhang Q., Yang Y. (2024). Naringenin ameliorates amyloid-β pathology and neuroinflammation in Alzheimer’s disease. Commun. Biol..

[32] Lee N., Youn K., Yoon J.H., Lee B., Kim D.H., Jun M. (2023). The Role of Fucoxanthin as a Potent Nrf2 Activator via Akt/GSK-3β/Fyn Axis against Amyloid-β Peptide-Induced Oxidative Damage. Antioxidants.

[33] Khan S., Ahmad K., Alshammari E.M., Adnan M., Baig M.H., Lohani M., Somvanshi P., Haque S. (2015). Implication of Caspase-3 as a Common Therapeutic Target for Multineurodegenerative Disorders and Its Inhibition Using Nonpeptidyl Natural Compounds. BioMed Res. Int..

[34] García-Revilla J., Ruiz R., Espinosa-Oliva A.M., Santiago M., García-Domínguez I., Camprubí-Ferrer L., Bachiller S., Deierborg T., Joseph B., de Pablos R.M. (2024). Dopaminergic neurons lacking Caspase-3 avoid apoptosis but undergo necrosis after MPTP treatment inducing a Galectin-3-dependent selective microglial phagocytic response. Cell Death Dis..

[35] Blandini F., Sinforiani E., Pacchetti C., Samuele A., Bazzini E., Zangaglia R., Nappi G., Martignoni E. (2006). Peripheral proteasome and caspase activity in Parkinson disease and Alzheimer disease. Neurology.

[36] Xu M., Wang X., Zhang Y., Ji N., Wang Q., Zhao T., Zhou C., Jia C. (2024). Profiling of the Proteins Interacting with Amyloid Beta Peptides in Clinical Samples by PACTS-TPP. J. Am. Soc. Mass Spectrom..

[37] Kommaddi R.P., Gowaikar R., P A H., Diwakar L., Singh K., Mondal A. (2024). Akt activation ameliorates deficits in hippocampal-dependent memory and activity-dependent synaptic protein synthesis in an Alzheimer’s disease mouse model. J. Biol. Chem..

[38] Wawrzyniak A., Krawczyk-Marć I., Żuryń A., Walocha J., Balawender K. (2025). Diversity, Functional Complexity, and Translational Potential of Glial Cells in the Central Nervous System. Int. J. Mol. Sci..

[39] Rostami J., Mothes T., Kolahdouzan M., Eriksson O., Moslem M., Bergström J., Ingelsson M., O’Callaghan P., Healy L.M., Falk A. (2021). Crosstalk between astrocytes and microglia results in increased degradation of α-synuclein and amyloid-β aggregates. J. Neuroinflamm..

[40] Kim H.s., Kim S., Shin S.J., Park Y.H., Nam Y., Kim C.w., Lee K.w., Kim S.-M., Jung I.D., Yang H.D. (2021). Gram-negative bacteria and their lipopolysaccharides in Alzheimer’s disease: Pathologic roles and therapeutic implications. Transl. Neurodegener..

[41] Brown G.C., Camacho M., Williams-Gray C.H. (2023). The Endotoxin Hypothesis of Parkinson’s Disease. Mov. Disord..

[42] Skrzypczak-Wiercioch A., Sałat K. (2022). Lipopolysaccharide-Induced Model of Neuroinflammation: Mechanisms of Action, Research Application and Future Directions for Its Use. Molecules.

[43] Kalyan M., Tousif A.H., Sonali S., Vichitra C., Sunanda T., Praveenraj S.S., Ray B., Gorantla V.R., Rungratanawanich W., Mahalakshmi A.M. (2022). Role of Endogenous Lipopolysaccharides in Neurological Disorders. Cells.

[44] Taherkhani A., Khodadadi P., Samie L., Azadian Z., Bayat Z. (2023). Flavonoids as Strong Inhibitors of MAPK3: A Computational Drug Discovery Approach. Int. J. Anal. Chem..

[45] Vaziri-Amjad S., Moradi-Najmi M., Taherkhani A. (2023). Natural Anthraquinones as Promising MAPK3 Inhibitors for Complementary Cancer Therapy. J. Chem..

[46] Li C., Zhuo C., Ma X., Li R., Chen X., Li Y., Zhang Q., Yang L., Wang L. (2024). Exploring the molecular targets of fingolimod and siponimod for treating the impaired cognition of schizophrenia using network pharmacology and molecular docking. Schizophrenia.

[47] Liu X., Peng X., Lin Z. (2021). Evodiamine Enhanced the Anti-Inflammation Effect of Clindamycin in the BEAS-2B Cells Infected with H5N1 and Pneumoniae D39 Through CREB-C/EBPβ Signaling Pathway. Viral Immunol..

[48] Yuan S.M., Gao K., Wang D.M., Quan X.Z., Liu J.N., Ma C.M., Qin C., Zhang L.F. (2011). Evodiamine improves congnitive abilities in SAMP8 and APP(swe)/PS1(ΔE9) transgenic mouse models of Alzheimer’s disease. Acta Pharmacol. Sin..

[49] Zhang Y., Wang J., Wang C., Li Z., Liu X., Zhang J., Lu J., Wang D. (2018). Pharmacological Basis for the Use of Evodiamine in Alzheimer’s Disease: Antioxidation and Antiapoptosis. Int. J. Mol. Sci..

[50] Kim Y.W., Zhao R.J., Park S.J., Lee J.R., Cho I.J., Yang C.H., Kim S.G., Kim S.C. (2008). Anti-inflammatory effects of liquiritigenin as a consequence of the inhibition of NF-kappaB-dependent iNOS and proinflammatory cytokines production. Br. J. Pharmacol..

[51] Li L., Fang H., Yu Y.H., Liu S.X., Yang Z.Q. (2021). Liquiritigenin attenuates isoprenaline-induced myocardial fibrosis in mice through the TGF-β1/Smad2 and AKT/ERK signaling pathways. Mol. Med. Rep..

[52] Valenzuela-Arzeta I.E., Soto-Rojas L.O., Flores-Martinez Y.M., Delgado-Minjares K.M., Gatica-Garcia B., Mascotte-Cruz J.U., Nava P., Aparicio-Trejo O.E., Reyes-Corona D., Martínez-Dávila I.A. (2023). LPS Triggers Acute Neuroinflammation and Parkinsonism Involving NLRP3 Inflammasome Pathway and Mitochondrial CI Dysfunction in the Rat. Int. J. Mol. Sci..

[53] Shademan B., Yousefi H., Sharafkhani R., Nourazarian A. (2025). LPS-Induced Neuroinflammation Disrupts Brain-Derived Neurotrophic Factor and Kinase Pathways in Alzheimer’s Disease Cell Models. Cell Mol. Neurobiol..

[54] Schnider T.W., Minto C.F., Luginbühl M., Egan T.D. (2022). The drug titration paradox: More drug does not correlate with more effect in individual clinical data. Br. J. Anaesth..

[55] Kazemi K., Fadl A., Sperandio F.F., Leask A. (2025). The Areca Nut and Oral Submucosal Fibrosis: A Narrative Review. Dent. J..

[56] Sharma M., Sarode S.C., Sarode G., Radhakrishnan R. (2024). Areca nut-induced oral fibrosis—Reassessing the biology of oral submucous fibrosis. J. Oral Biosci..

[57] Caesar L.K., Cech N.B. (2019). Synergy and antagonism in natural product extracts: When 1 + 1 does not equal 2. Nat. Prod. Rep..

[58] Vaou N., Stavropoulou E., Voidarou C.C., Tsakris Z., Rozos G., Tsigalou C., Bezirtzoglou E. (2022). Interactions between Medical Plant-Derived Bioactive Compounds: Focus on Antimicrobial Combination Effects. Antibiotics.

[59] Prasansuklab A., Sukjamnong S., Theerasri A., Hu V.W., Sarachana T., Tencomnao T. (2023). Transcriptomic analysis of glutamate-induced HT22 neurotoxicity as a model for screening anti-Alzheimer’s drugs. Sci. Rep..

[60] Prasertsuksri P., Kraokaew P., Pranweerapaiboon K., Sobhon P., Chaithirayanon K. (2023). Neuroprotection of Andrographolide against Neurotoxin MPP(+)-Induced Apoptosis in SH-SY5Y Cells via Activating Mitophagy, Autophagy, and Antioxidant Activities. Int. J. Mol. Sci..

[61] Can Ağca A., Altay D., Kul H., Ceylan A.F., Sever Yilmaz B. (2025). *Moltkia coerulea* extracts alleviate caspase-3 activity via reducing oxidative stress in LPS-induced neurotoxicity in BV-2 cells. Turk. J. Med. Sci..

[62] Ma J., Motsinger-Reif A. (2019). Current Methods for Quantifying Drug Synergism. Proteome Bioinform..

[63] Duarte D., Vale N. (2022). Evaluation of synergism in drug combinations and reference models for future orientations in oncology. Curr. Res. Pharmacol. Drug Discov..

[64] Gao X., Li S., Cong C., Wang Y., Xu L. (2021). A Network Pharmacology Approach to Estimate Potential Targets of the Active Ingredients of Epimedium for Alleviating Mild Cognitive Impairment and Treating Alzheimer’s Disease. Evid. Based Complement. Altern. Med..

[65] Liang P., Wang Y., Liu J., Huang H., Li Y., Kang J., Li G., Wu H. (2025). Identification and Exploration of Immunity-Related Genes and Natural Products for Alzheimer’s Disease Based on Bioinformatics, Molecular Docking, and Molecular Dynamics. Immun. Inflamm. Dis..

[66] Malar D.S., Verma K., Prasanth M.I., Tencomnao T., Brimson J.M. (2024). Network analysis-guided drug repurposing strategies targeting LPAR receptor in the interplay of COVID, Alzheimer’s, and diabetes. Sci. Rep..

[67] Hu M., Yan H., Li H., Feng Y., Sun W., Ren Y., Ma L., Zeng W., Huang F., Jiang Z. (2023). Use of network pharmacology and molecular docking to explore the mechanism of action of curcuma in the treatment of osteosarcoma. Sci. Rep..

[68] Shan C., Ji X., Wu Z., Zhao J. (2022). Network pharmacology combined with GEO database identifying the mechanisms and molecular targets of Polygoni Cuspidati Rhizoma on Peri-implants. Sci. Rep..

[69] Chhotaray S., Verma K., Badgayan N.D., Banerjee R., Jal S. Identification of Hub Genes Indicating Association of Atherosclerosis with Rheumatoid Arthritis. Proceedings of the 2024 15th International Conference on Computing Communication and Networking Technologies (ICCCNT).

[70] Tunyasuvunakool K., Adler J., Wu Z., Green T., Zielinski M., Žídek A., Bridgland A., Cowie A., Meyer C., Laydon A. (2021). Highly accurate protein structure prediction for the human proteome. Nature.

[71] Shivanika C., Kumar Deepak S., Ragunathan V., Tiwari P., Sumitha A., Brindha Devi P. (2022). Molecular docking, validation, dynamics simulations, and pharmacokinetic prediction of natural compounds against the SARS-CoV-2 main-protease. J. Biomol. Struct. Dyn..

[72] Forli S., Huey R., Pique M.E., Sanner M.F., Goodsell D.S., Olson A.J. (2016). Computational protein-ligand docking and virtual drug screening with the AutoDock suite. Nat. Protoc..

[73] Terefe E.M., Ghosh A. (2022). Molecular Docking, Validation, Dynamics Simulations, and Pharmacokinetic Prediction of Phytochemicals Isolated From *Croton dichogamus* Against the HIV-1 Reverse Transcriptase. Bioinform. Biol. Insights.

[74] Roomi M.S., Culletta G., Longo L., Filgueira de Azevedo W., Perricone U., Tutone M. (2025). Docking in the Dark: Insights into Protein-Protein and Protein-Ligand Blind Docking. Pharmaceuticals.

[75] Janpaijit S., Sillapachaiyaporn C., Theerasri A., Charoenkiatkul S., Sukprasansap M., Tencomnao T. (2023). *Cleistocalyx nervosum* var. *paniala* Berry Seed Protects against TNF-α-Stimulated Neuroinflammation by Inducing HO-1 and Suppressing NF-κB Mechanism in BV-2 Microglial Cells. Molecules.

