# Supplementary material for: Exploring Mechanistic Targets of Areca catechu Against Neurodegenerative Diseases Through an Integrated Network Pharmacology, Molecular Docking, and Experimental Approaches
Source: Int J Mol Sci. 2026 Jun 7;27(12):5169. doi: 10.3390/ijms27125169 (PMC13300171; doi:10.3390/ijms27125169)
Supplement: Supplementary file 1 [file ijms-27-05169-s001.zip › ijms-4259197-supplementary.pdf]

Supplementary Materials

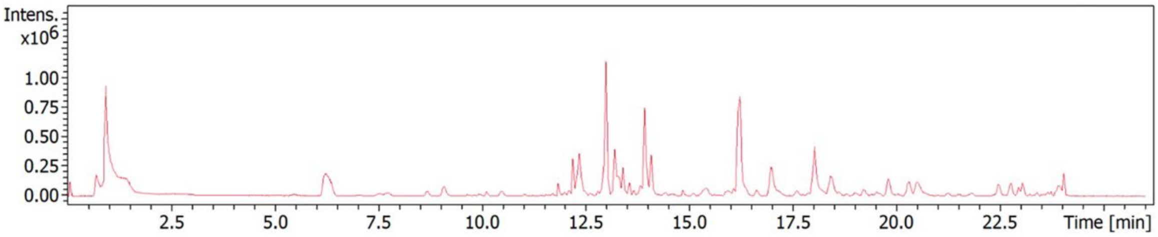

Figure S1. LC-MS chromatogram for ACEA.

Table S1. Tentatively identified phytocompounds in ACEA by LC-QTOF analysis.

NO	Compound name	Molecular weight	Molecular Formula	Retention time (min)	*m/z*	ppm
1	Arecoline	155.09468	$C_8H_{13}NO_2$	0.91	156.10197 (M+H+)	-0.26
2	Evocarpine	339.25637	$C_{23}H_{33}NO$	12.34	340.25768 (M+H+)	17.21
3	Magnoshinin	414.20436	$C_{24}H_{30}O_6$	13.25	415.21027 (M+H+)	3.07
4	Montanol	352.26152	$C_{21}H_{36}O_4$	15.93	375.25001 (M+Na+)	3.18
5	(+)-Eudesmin	386.17304	$C_{22}H_{26}O_6$	13.41	387.19122 (M+H+)	-28.45
6	Buddledin A	276.17265	$C_{17}H_{24}O_3$	13.56	277.17888 (M+H+)	3.50
7	Perlolyrine	264.08992	$C_{16}H_{12}N_2O_2$	12.79	265.09648 (M+H+)	2.64
8	Podecdysone B	462.29832	$C_{27}H_{42}O_6$	15.61	485.28756 (M+Na+)	0.76
9	quinic acid	192.06342	$C_7H_{12}O_6$	0.84	215.05297 (M+Na+)	0.99
10	Pycnolide	364.1887	$C_{20}H_{28}O_6$	12.99	387.17933 (M+Na+)	-2.66
11	senkyunolide B	204.07869	$C_{12}H_{12}O_3$	13.93	205.08536 (M+H+)	2.87
12	4-Methylthio-2-oxobutanoic acid	148.01944	$C_5H_8O_3S$	13.93	149.02287 (M+H+)	25.99
13	2-Palmitoylglycerol	330.5	$C_{19}H_{38}O_4$	18.03	331.28329 (M+H+)	3.09
14	(2S)-Flavanone	224.08378	$C_{15}H_{12}O_2$	13.2	225.09048 (M+H+)	2.46
15	Catechin	290.07908	$C_{15}H_{14}O_6$	6.21	291.08594	1.38

					(M+H+)	
16	Theobromine	180.06474	$C_7H_8N_4O_2$	0.7	203.05274 (M+Na+)	9.76
17	Agroclavine	238.14708	$C_{16}H_{18}N_2$	13.3	239.16099 (M+H+)	-28.12
18	Crotanecine	171.08959	$C_8H_{13}NO_3$	0.92	172.09697 (M+H+)	-0.74
19	Flavone	222.06812	$C_{15}H_{10}O_2$	0.88	245.06335 (M+Na+)	-24.77
20	Cucurbitacin E	556.3038	$C_{32}H_{44}O_8$	13.93	579.29026 (M+Na+)	5.63
21	Precocene II	220.11001	$C_{13}H_{16}O_3$	13.56	221.11666 (M+H+)	2.66
22	2,6-Nonadienal	138.10453	$C_9H_{14}O$	13.56	161.09566 (M+Na+)	-10.33
23	Solanocapsine	430.35614	$C_{27}H_{46}N_2O_2$	12.43	453.34121 (M+Na+)	10.44
24	Pterostilbene	256.11001	$C_{16}H_{16}O_3$	13.2	279.0984 (M+Na+)	5.14
25	Citronellyl acetate	198.16208	$C_{12}H_{22}O_2$	13.3	199.16886 (M+H+)	2.12
26	Vasconine	266.11817	$C_{17}H_{16}NO_2$	12.99	267.12199 (M+H+)	12.83
27	Octanal	128.12019	$C_8H_{16}O$	13.3	129.12698 (M+H+)	3.40
28	Farnesylacetone	262.4	$C_{18}H_{30}O$	16.97	263.23545 (M+H+)	5.78
29	Dehydroabietic acid	300.20906	$C_{20}H_{28}O_2$	19.19	301.21011 (M+H+)	20.40
30	Estriol	288.17265	$C_{18}H_{24}O_3$	13.46	311.16306 (M+Na+)	-2.59
31	Lactucin	276.09983	$C_{15}H_{16}O_5$	12.09	277.10391 (M+H+)	11.47
32	L-Glutamine	146.06917	$C_5H_{10}N_2O_3$	13.67	147.07953 (M+H+)	-21.14
33	Cryptolepine	232.1001	$C_{16}H_{12}N_2$	14.84	255.08309 (M+Na+)	29.00
34	Tremetone	202.09944	$C_{13}H_{14}O_2$	13.56	203.10627 (M+H+)	2.03
35	Phellodendrine	342.42	$C_{20}H_{24}NO_4$	0.91	343.1865 (M+H+)	-25.34
36	Cimifugin	306.1104	$C_{16}H_{18}O_6$	10.47	307.11397 (M+H+)	11.99
37	Lathyrine	182.0804	$C_7H_{10}N_4O_2$	0.88	205.07096 (M+Na+)	-4.47
38	Carapanaubine	428.19484	$C_{23}H_{28}N_2O_6$	13.15	429.19062 (M+H+)	26.68
39	Lucidine B	467.38779	$C_{30}H_{49}N_3O$	16.17	468.4006 (M+H+)	-12.29
40	Abietal	286.22981	$C_{20}H_{30}O$	12.49	309.22611	-23.34

					(M+Na+)	
41	Methyl Anthranilate	151.06336	$C_8H_9NO_2$	0.88	152.07075 (M+H+)	-0.80
42	Thalsimine	636.28371	$C_{38}H_{40}N_2O_7$	18.6	637.30192 (M+H+)	-17.40
43	Santin	344.08965	$C_{18}H_{16}O_7$	13.16	345.09562 (M+H+)	3.74
44	Puerarin	416.11079	$C_{21}H_{20}O_9$	10.1	439.09827 (M+Na+)	5.37
45	Undecanoic acid	186.16208	$C_{11}H_{22}O_2$	13.2	209.15345 (M+Na+)	-9.14
46	Conessine	356.31934	$C_{24}H_{40}N_2$	19.52	379.31708 (M+Na+)	-22.93
47	Cassine	297.26694	$C_{18}H_{35}NO_2$	17.99	298.27352 (M+H+)	1.88
48	Coniferaldehyde	178.06303	$C_{10}H_{10}O_3$	15.87	179.07016 (M+H+)	0.76
49	Erythrocentaurin	176.04737	$C_{10}H_8O_3$	12.83	177.0544 (M+H+)	1.39
50	Pfaffic acid	440.32925	$C_{29}H_{44}O_3$	14.91	441.32749 (M+H+)	20.12
51	Eburnamonine	294.17331	$C_{19}H_{22}N_2O$	13.56	317.17106 (M+Na+)	-27.47
52	Lanceotoxin A	620.28344	$C_{32}H_{44}O_{12}$	17.03	621.30823 (M+H+)	-28.47
53	4-Hydroxy-4-(3-hydroxy-1-butenyl)-3,5,5-trimethyl-2-cyclohexen-1-one	224.14133	$C_{13}H_{20}O_3$	12.99	225.14796 (M+H+)	2.61
54	Austrobailignan 1	382.10531	$C_{21}H_{18}O_7$	0.7	383.11575 (M+H+)	-8.36
55	Erucic acid	338.31868	$C_{22}H_{42}O_2$	19.52	339.32442 (M+H+)	4.02
56	Eutypine	186.06812	$C_{12}H_{10}O_2$	12.83	209.05886 (M+Na+)	-5.43
57	Phytosphingosine	317.29317	$C_{18}H_{39}NO_3$	15.1	340.281 (M+Na+)	5.56
58	Methylitaconate	144.04228	$C_6H_8O_4$	0.71	145.04969 (M+H+)	-0.90
59	Pyridoxine	169.07393	$C_8H_{11}NO_3$	7.52	170.08107 (M+H+)	0.73
60	Gambiriin A3	580.15816	$C_{30}H_{28}O_{12}$	8.66	603.14569 (M+Na+)	3.72
61	Petroselinic Acid	282.25604	$C_{18}H_{34}O_2$	19.6	305.24409 (M+Na+)	5.53
62	Disinomenine	656.30994	$C_{38}H_{44}N_2O_8$	16.24	657.3163 (M+H+)	1.17
63	Dimethamine	408.25266	$C_{24}H_{32}N_4O_2$	19.78	431.24321 (M+Na+)	-2.24
64	Narcissin	624.5	$C_{28}H_{32}O_{16}$	12.13	625.1746	2.79

					(M+H+)	
65	Thiarubrine A	228.00678	$C_{13}H_8S_2$	13.04	251.00022 (M+Na+)	-16.25
66	Phenylacetaldehyde	120.05755	C_8H_8O	12.99	121.06446 (M+H+)	2.96
67	Homostachydrine	157.11034	$C_8H_{15}NO_2$	0.91	158.11514 (M+H+)	15.54
68	Dihydroshikonofuran	260.14133	$C_{16}H_{20}O_3$	19.8	261.14797 (M+H+)	2.21
69	Ginkgolide C	440.13193	$C_{20}H_{24}O_{11}$	12.23	441.14982 (M+H+)	-24.22
70	1,2,3,4-Tetrahydro-Beta-Carboline-3-Carboxylic Acid	216.24	$C_{12}H_{12}N_2O_2$	10.43	239.07849 (M+Na+)	5.36
71	N-Ethylacetamide	87.06845	C_4H_9NO	0.96	110.06002 (M+Na+)	-21.10
72	Mannitol	182.07908	$C_6H_{14}O_6$	0.69	205.06771 (M+Na+)	6.04
73	Cinnamic acid	148.05246	$C_9H_8O_2$	13.25	149.05933 (M+H+)	2.70
74	Mallotochromene	442.16286	$C_{24}H_{26}O_8$	11.01	443.16646 (M+H+)	8.17
75	Kaurenoic Acid	302.22472	$C_{20}H_{30}O_2$	17.98	303.22788 (M+H+)	13.25
76	Tiglyl tiglate	168.1151	$C_{10}H_{16}O_2$	9.93	169.11969 (M+H+)	15.71
77	4'-O-Methylxanthohumol	368.16247	$C_{22}H_{24}O_5$	13.3	369.16868 (M+H+)	2.70
78	delta-Tocotrienol	396.30302	$C_{27}H_{40}O_2$	14.88	397.30143 (M+H+)	21.97
79	Broussonin C	312.17265	$C_{20}H_{24}O_3$	15.86	313.17635 (M+H+)	11.20
80	Oleamide	281.27203	$C_{18}H_{35}NO$	17.74	304.25933 (M+Na+)	8.20
81	Dehydrocurdione	234.16208	$C_{15}H_{22}O_2$	13.56	235.16852 (M+H+)	3.25
82	Cinchonamine	296.18897	$C_{19}H_{24}N_2O$	14.43	297.20225 (M+H+)	-20.57
83	Taxodione	314.18831	$C_{20}H_{26}O_3$	13.72	315.19422 (M+H+)	4.06
84	Flindersine	227.09468	$C_{14}H_{13}NO_2$	12.59	228.10139 (M+H+)	2.37
85	Naphthalene-1,2-diol	160.05246	$C_{10}H_8O_2$	15.87	161.05934 (M+H+)	2.44
86	Deoxytubulosine	459.28874	$C_{29}H_{37}N_3O_2$	14.43	460.29156 (M+H+)	9.41
87	Oxolucidine B	483.3827	$C_{30}H_{49}N_3O_2$	14.64	484.38338 (M+H+)	13.24
88	Silandrin	466.12645	$C_{25}H_{22}O_9$	0.8	467.13651 (M+H+)	-6.06

89	Viguiestenin	392.18361	$C_{21}H_{28}O_7$	13.77	393.18014 (M+H+)	27.20
90	Ecgonone methyl ester	197.10525	$C_{10}H_{15}NO_3$	11.67	198.11215 (M+H+)	1.74
91	Hydnocarpic acid	252.20906	$C_{16}H_{28}O_2$	15.96	253.2131 (M+H+)	12.42
92	Neral	152.12019	$C_{10}H_{16}O$	13.2	153.12685 (M+H+)	3.72
93	2-Palmitoleoyl-glycerol	350.24586	$C_{21}H_{34}O_4$	16.31	351.24867 (M+H+)	12.40
94	Aspidinol	224.10492	$C_{12}H_{16}O_4$	12.64	225.10922 (M+H+)	13.12
95	16-Dehydropregnenolone	314.22472	$C_{21}H_{30}O_2$	17.86	315.23128 (M+H+)	1.91
96	Gingerdione	292.16756	$C_{17}H_{24}O_4$	12.6	293.17223 (M+H+)	8.66
97	Sebacic acid	202.12058	$C_{10}H_{18}O_4$	12.66	203.12592 (M+H+)	9.36
98	(-)-Salsoline	193.11034	$C_{11}H_{15}NO_2$	13.14	194.11714 (M+H+)	2.27
99	Pterosterone	480.30889	$C_{27}H_{44}O_7$	14.9	481.30737 (M+H+)	17.99
100	Furfuryl alcohol	98.0368	$C_5H_6O_2$	13.93	121.02816 (M+Na+)	-16.43
101	Tetryl	169.14675	$C_{10}H_{19}NO$	12.79	192.1377 (M+Na+)	-7.49
102	Adifoline	424.12711	$C_{22}H_{20}N_2O_7$	0.79	425.12685 (M+H+)	17.70
103	butyric acid	88.05246	$C_4H_8O_2$	13.3	89.05953 (M+H+)	2.27
104	Sphinganine	301.29826	$C_{18}H_{39}NO_2$	16.27	324.28855 (M+Na+)	-2.33
105	Isodomedin	392.22002	$C_{22}H_{32}O_6$	16.93	393.23854 (M+H+)	-28.95
106	Ellipticine	246.11576	$C_{17}H_{14}N_2$	15.88	269.09895 (M+Na+)	26.50
107	Flindersine	227.09468	$C_{14}H_{13}NO_2$	15.42	250.08539 (M+Na+)	-4.37
108	Apiforol	274.08417	$C_{15}H_{14}O_5$	11.45	275.09128 (M+H+)	0.53
109	Cornudentanone	378.24077	$C_{22}H_{34}O_5$	15.42	379.23664 (M+H+)	29.85
110	Phytuberin	294.18322	$C_{17}H_{26}O_4$	13.13	295.18989 (M+H+)	1.77

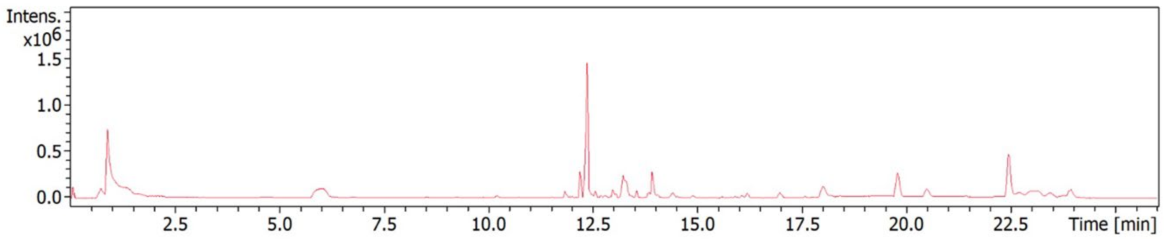

Figure S2. LC-MS chromatogram for ACEE.

Table S2. Tentatively identified phytocompounds in ACEE by LC-QTOF analysis.

NO	Compound name	Molecular weight	Molecular Formula	Retention time (min)	*m/z*	ppm
1	Arecoline	155.0947	$C_8H_{13}NO_2$	0.9	156.1021 (M+H+)	-1.10
2	Evocarpine	339.2564	$C_{23}H_{33}NO$	12.32	340.2603 (M+H+)	9.60
3	Magnoshinin	414.2044	$C_{24}H_{30}O_6$	13.23	415.2131 (M+H+)	-3.73
4	Montanol	352.2615	$C_{21}H_{36}O_4$	18	353.2674 (M+H+)	3.61
5	(+)-Eudesmin	386.1730	$C_{22}H_{26}O_6$	12.96	387.1810 (M+H+)	-1.94
6	Buddledin A	276.1727	$C_{17}H_{24}O_3$	13.55	277.1805 (M+H+)	-2.19
7	Perlolyrine	264.0899	$C_{16}H_{12}N_2O_2$	12.79	265.0971 (M+H+)	0.30
8	Podecdysone B	462.2983	$C_{27}H_{42}O_6$	15.6	485.2909 (M+Na+)	-6.52
9	quinic acid	192.0634	$C_7H_{12}O_6$	0.68	193.0707 (M+H+)	-0.05
10	Erucamide	337.6	$C_{22}H_{43}NO$	22.44	338.3421 (M+H+)	-0.88
11	Evodiamine	303.1372	$C_{19}H_{17}N_3O$	0.9	304.1395 (M+H+)	16.41
12	Arecaidine	141.0790	$C_7H_{11}NO_2$	0.72	142.0864 (M+H+)	-0.79
13	Melochinone	331.1573	$C_{22}H_{21}NO_2$	6.05	332.1708 (M+H+)	-19.04
14	[10]-Gingerol	350.2459	$C_{21}H_{34}O_4$	12.32	351.2514 (M+H+)	4.49
15	Linamarin	247.1056	$C_{10}H_{17}NO_6$	0.9	270.0952 (M+Na+)	0.48
16	Cucurbitacin A	574.3144	$C_{32}H_{46}O_9$	13.55	575.3322 (M+H+)	-18.68
17	Cassyfiline	341.1264	$C_{19}H_{19}NO_5$	0.69	342.1402	-19.28

					(M+H+)	
18	Symlandine	381.2153	$C_{20}H_{31}NO_6$	12.96	404.2056 (M+Na+)	-1.84
19	Sclareol	308.2717	$C_{20}H_{36}O_2$	21.42	309.2800 (M+H+)	-3.93
20	Pithecolobine	382.3674	$C_{22}H_{46}N_4O$	23.93	383.3675 (M+H+)	18.17
21	Danielone	212.0685	$C_{10}H_{12}O_5$	10.2	213.0745 (M+H+)	5.92
22	Gondoic acid	310.2874	$C_{20}H_{38}O_2$	22.93	311.2957 (M+H+)	-4.00
23	Crinamine	301.1315	$C_{17}H_{19}NO_4$	12.69	324.1219 (M+Na+)	-2.26
24	Lysergamide	267.1372	$C_{16}H_{17}N_3O$	0.73	290.1240 (M+Na+)	11.06
25	Isorhynchophylline	384.2050	$C_{22}H_{28}N_2O_4$	12.55	385.2078 (M+H+)	11.51
26	Trigonelline	137.1400	$C_7H_7NO_2$	0.73	138.0551 (M+H+)	-1.18
27	Cassaidine	407.3037	$C_{24}H_{41}NO_4$	19.78	408.3128 (M+H+)	-4.79
28	Calafatimine	636.2837	$C_{38}H_{40}N_2O_7$	16.19	659.2883 (M+Na+)	-23.55
29	stachydrine	143.1800	$C_7H_{13}NO_2$	0.75	144.1021 (M+H+)	-0.92
30	5-Hydroxymethylfurfural	126.0317	$C_6H_6O_3$	0.9	127.0393 (M+H+)	-2.18
31	procurcumenol	234.33	$C_{15}H_{22}O_2$	13.58	235.1701 (M+H+)	-3.50
32	2(5H)-Furanone	84.0211	$C_4H_4O_2$	0.9	85.0286 (M+H+)	-1.59
33	Vernoflexuoside	408.1785	$C_{21}H_{28}O_8$	13.38	409.1767 (M+H+)	22.18
34	Pheophytin a	870.5663	$C_{55}H_{74}N_4O_5$	23.98	871.5719 (M+H+)	1.55
35	Vanillyl alcohol	154.0630	$C_8H_{10}O_3$	12.1	155.0709 (M+H+)	-5.04
36	homoarecoline	169.2209	$C_9H_{15}NO_2$	0.85	170.1179 (M+H+)	-1.90
37	decanoic acid	172.26	$C_{10}H_{20}O_2$	13.19	195.1394 (M+Na+)	-19.19
38	stearic acid	284.5000	$C_{18}H_{36}O_2$	19.94	307.2645 (M+Na+)	-11.27
39	myristoleic acid	226.3550	$C_{14}H_{26}O_2$	13.63	249.1869 (M+Na+)	-17.04
40	alpha- terpineol	154.2500	$C_{10}H_{18}O$	13.2	177.1294 (M+Na+)	-25.10
41	ethyl nicotinate	151.1600	$C_8H_9NO_2$	0.89	152.0700 (M+H+)	4.17
42	3-carene	136.23	$C_{10}H_{16}$	12.66	159.1182	-14.29

					(M+Na+)	
43	lauric acid	200.3200	$C_{12}H_{24}O_2$	12.95	223.1702 (M+Na+)	-18.18
44	epicatechin	290.2700	$C_{15}H_{14}O_6$	6.16	291.0892 (M+H+)	-9.86
45	liquiritigenin	256.25	$C_{15}H_{12}O_4$	0.73	279.0707 (M+Na+)	-28.79
46	tetradecanoic acid	228.3700	$C_{14}H_{28}O_2$	14.57	251.1970 (M+Na+)	7.46

Table S3. Lists of Alzheimer's disease (AD)-related targets, Parkinson's disease (PD)-related targets, and common disease targets in both AD and PD.

NO.	Target name	Disease	NO.	Target name	Disease
1	APP	AD	274	LRPAP1	AD
2	SNCA	AD	275	HSPA1B	AD
3	LRRK2	AD	276	PNMT	AD
4	VPS35	AD	277	NCF2	AD
5	PSEN1	AD	278	TCN2	AD
6	PINK1	AD	279	AMFR	AD
7	PARK7	AD	280	TALDO1	AD
8	APOE	AD	281	FBP1	AD
9	MAPT	AD	282	SLC30A10	AD
10	PSEN2	AD	283	RPS8	AD
11	GRN	AD	284	RPL14	AD
12	ACE	AD	285	RPL6	AD
13	CLU	AD	286	RPL23A	AD
14	INS	AD	287	SLC38A2	AD
15	TREM2	AD	288	PTGIS	AD
16	GDNF	AD	289	SNCA	PD
17	ACHE	AD	290	LRRK2	PD
18	ABCA7	AD	291	VPS35	PD
19	GSK3B	AD	292	PINK1	PD
20	BDNF	AD	293	PARK7	PD
21	IGF1	AD	294	GDNF	PD
22	IL1B	AD	295	MAPT	PD
23	TNF	AD	296	BDNF	PD
24	MAOB	AD	297	MAOB	PD
25	BCHE	AD	298	NR4A2	PD
26	BACE1	AD	299	SLC6A3	PD
27	AGER	AD	300	SLC18A2	PD
28	SORL1	AD	301	DDC	PD
29	GRIN2B	AD	302	ATP13A2	PD
30	NR4A2	AD	303	TH	PD

31	CSF1R	AD	304	SOD1	PD
32	SLC6A3	AD	305	DRD2	PD
33	PPARG	AD	306	S100B	PD
34	SLC18A2	AD	307	IL1B	PD
35	DDC	AD	308	SYNJ1	PD
36	ATP13A2	AD	309	MAPK3	PD
37	VEGFA	AD	310	AKT1	PD
38	TH	AD	311	GSTP1	PD
39	CYP46A1	AD	312	GSTM1	PD
40	MME	AD	313	PPARGC1A	PD
41	NTF3	AD	314	NOS1	PD
42	NOS3	AD	315	HSPA9	PD
43	APOC1	AD	316	FBXO7	PD
44	PLAU	AD	317	UCHL1	PD
45	CD33	AD	318	DNAJC6	PD
46	PICALM	AD	319	CYP2D6	PD
47	SOD1	AD	320	HMOX1	PD
48	BIN1	AD	321	TNF	PD
49	IDE	AD	322	NGF	PD
50	LEP	AD	323	IL6	PD
51	DRD2	AD	324	ABCB1	PD
52	IGFBP3	AD	325	GFAP	PD
53	HTR6	AD	326	MAOA	PD
54	ADAM17	AD	327	INS	PD
55	IL4	AD	328	EPO	PD
56	CHAT	AD	329	VPS13C	PD
57	S100B	AD	330	SOD2	PD
58	IL1A	AD	331	HLA-DRA	PD
59	ICAM1	AD	332	DRD1	PD
60	CDK5	AD	333	IGF2	PD
61	FYN	AD	334	BST1	PD
62	SYNJ1	AD	335	DNAJC13	PD
63	UCHL1	AD	336	SNCB	PD
64	MAPK3	AD	337	CYP1A1	PD
65	GCG	AD	338	HNMT	PD
66	TLR4	AD	339	NAT2	PD
67	AKT1	AD	340	SLC11A2	PD
68	TTR	AD	341	HTT	PD
69	IL10	AD	342	POLG	PD
70	HFE	AD	343	DBH	PD
71	MPO	AD	344	PARP1	PD
72	A2M	AD	345	MAPK1	PD
73	GSTP1	AD	346	TARDBP	PD
74	ADAM10	AD	347	HTRA2	PD

75	ESR1	AD	348	ANG	PD
76	NCSTN	AD	349	TFAM	PD
77	GSTM1	AD	350	HSPA8	PD
78	PPARGC1A	AD	351	CP	PD
79	ENO1	AD	352	HFE	PD
80	PRNP	AD	353	NQO1	PD
81	SOD2	AD	354	ALDH2	PD
82	NOS1	AD	355	SNCAIP	PD
83	EIF2S1	AD	356	HSPA1A	PD
84	HSPA9	AD	357	DNM1L	PD
85	ABCA1	AD	358	CYP2E1	PD
86	ESR2	AD	359	GAK	PD
87	FBXO7	AD	360	FGB	PD
88	DNAJC6	AD	361	TMEM230	PD
89	LDLR	AD	362	VDR	PD
90	BACE2	AD	363	ESR2	PD
91	CYP2D6	AD	364	HLA-DRB1	PD
92	HMOX1	AD	365	PLA2G6	PD
93	NGF	AD	366	CASP3	PD
94	F2	AD	367	GSTT1	PD
95	IL6	AD	368	GIGYF2	PD
96	ABCB1	AD	369	IGF1R	PD
97	GFAP	AD	370	MTHFR	PD
98	MAOA	AD	371	TRPM2	PD
99	IL2	AD	372	NQO2	PD
100	EPO	AD	373	GRK6	PD
101	TOMM40	AD	374	GSTO1	PD
102	CD2AP	AD	375	KLK6	PD
103	VPS13C	AD	376	GSTO2	PD
104	EPHA1	AD	377	GDF5	PD
105	CST3	AD	378	CASP9	PD
106	WWOX	AD	379	CREB1	PD
107	IGF1R	AD	380	EN1	PD
108	INPP5D	AD	381	EPHX1	PD
109	ABI3	AD	382	CAST	PD
110	HLA-DRA	AD	383	BAG5	PD
111	DRD1	AD	384	MAP3K5	PD
112	BCL2	AD	385	RAB32	PD
113	NPY	AD	386	HGF	PD
114	PTK2B	AD	387	INSR	PD
115	INSR	AD	388	DDIT4	PD
116	CRH	AD	389	ENO2	PD
117	IGF2	AD	390	IGF2R	PD
118	BAX	AD	391	GSTA4	PD

119	CASP3	AD	392	MAPT-AS1	PD
120	RELN	AD	393	HMGCR	PD
121	PLCG2	AD	394	ATM	PD
122	BST1	AD	395	A2M	PD
123	CHRNA7	AD	396	NDUFV2	PD
124	APBB1	AD	397	EIF2AK2	PD
125	ECE1	AD	398	RFC1	PD
126	DNAJC13	AD	399	TNR	PD
127	SNCB	AD	400	MIR181C	PD
128	PPARA	AD	401	HBG1	PD
129	PON1	AD	402	MAP2	PD
130	ABAT	AD	403	AIF1	PD
131	NTRK2	AD	404	FCER2	PD
132	CYP1A1	AD	405	SNCA-AS1	PD
133	HNMT	AD	406	MTRR	PD
134	NGFR	AD	407	PHACTR2	PD
135	NAT2	AD	408	TNK2	PD
136	C3	AD	409	DRAXIN	PD
137	GLUL	AD	410	CNTNAP2	PD
138	HLA-DRB1	AD	411	MAG	PD
139	HSPA1A	AD	412	CEACAM6	PD
140	KLK6	AD	413	EDN1	PD
141	SLC11A2	AD	414	ADARB2	PD
142	HTT	AD	415	TCN2	PD
143	AGTR1	AD	416	FBP1	PD
144	CYP19A1	AD	417	SLC30A10	PD
145	CTSD	AD	418	MTA1	PD
146	NFE2L2	AD	419	RPL6	PD
147	CLOCK	AD	420	SLC38A2	PD
148	PARP1	AD	421	PINK1-AS	PD
149	LPL	AD	422	PTGIS	PD
150	EIF2AK2	AD	423	SNCA	AD-PD
151	POLG	AD	424	LRRK2	AD-PD
152	DBH	AD	425	VPS35	AD-PD
153	PPP2R2B	AD	426	PINK1	AD-PD
154	IRS1	AD	427	PARK7	AD-PD
155	LRP8	AD	428	GDNF	AD-PD
156	MAPK1	AD	429	MAPT	AD-PD
157	VLDLR	AD	430	BDNF	AD-PD
158	RCAN1	AD	431	MAOB	AD-PD
159	MT3	AD	432	NR4A2	AD-PD
160	TARDBP	AD	433	SLC6A3	AD-PD
161	HTRA2	AD	434	SLC18A2	AD-PD
162	ANG	AD	435	DDC	AD-PD

163	HMGCR	AD	436	ATP13A2	AD-PD
164	LRP1	AD	437	TH	AD-PD
165	TFAM	AD	438	SOD1	AD-PD
166	STAT3	AD	439	DRD2	AD-PD
167	GAPDH	AD	440	S100B	AD-PD
168	NTRK1	AD	441	IL1B	AD-PD
169	HSPA8	AD	442	SYNJ1	AD-PD
170	CP	AD	443	MAPK3	AD-PD
171	TF	AD	444	AKT1	AD-PD
172	MIR146A	AD	445	GSTP1	AD-PD
173	PILRA	AD	446	GSTM1	AD-PD
174	NQO1	AD	447	PPARGC1A	AD-PD
175	DPYSL2	AD	448	NOS1	AD-PD
176	MTHFR	AD	449	HSPA9	AD-PD
177	ALDH2	AD	450	FBXO7	AD-PD
178	SNCAIP	AD	451	UCHL1	AD-PD
179	VSNL1	AD	452	DNAJC6	AD-PD
180	DHCR24	AD	453	CYP2D6	AD-PD
181	DNM1L	AD	454	HMOX1	AD-PD
182	CYP2E1	AD	455	TNF	AD-PD
183	GAK	AD	456	NGF	AD-PD
184	ARC	AD	457	IL6	AD-PD
185	FGB	AD	458	ABCB1	AD-PD
186	TMEM230	AD	459	GFAP	AD-PD
187	VDR	AD	460	MAOA	AD-PD
188	APOA1	AD	461	INS	AD-PD
189	FAS	AD	462	EPO	AD-PD
190	PLA2G6	AD	463	VPS13C	AD-PD
191	GSTT1	AD	464	SOD2	AD-PD
192	GIGYF2	AD	465	HLA-DRA	AD-PD
193	TRPM2	AD	466	DRD1	AD-PD
194	KLC1	AD	467	IGF2	AD-PD
195	SERPINE2	AD	468	BST1	AD-PD
196	PON3	AD	469	DNAJC13	AD-PD
197	NQO2	AD	470	SNCB	AD-PD
198	IL6R	AD	471	CYP1A1	AD-PD
199	GSTO1	AD	472	HNMT	AD-PD
200	GSTO2	AD	473	NAT2	AD-PD
201	GDF5	AD	474	SLC11A2	AD-PD
202	CASP9	AD	475	HTT	AD-PD
203	APOA4	AD	476	POLG	AD-PD
204	CREB1	AD	477	DBH	AD-PD
205	EPHX1	AD	478	PARP1	AD-PD
206	GSS	AD	479	MAPK1	AD-PD

207	CAST	AD	480	TARDBP	AD-PD
208	CASS4	AD	481	HTRA2	AD-PD
209	GAPDHS	AD	482	ANG	AD-PD
210	CALM1	AD	483	TFAM	AD-PD
211	TSPAN14	AD	484	HSPA8	AD-PD
212	NCK2	AD	485	CP	AD-PD
213	CHRNB2	AD	486	HFE	AD-PD
214	TPP1	AD	487	NQO1	AD-PD
215	IQCK	AD	488	ALDH2	AD-PD
216	MS4A4A	AD	489	SNCAIP	AD-PD
217	BAG5	AD	490	HSPA1A	AD-PD
218	MAP3K5	AD	491	DNM1L	AD-PD
219	HGF	AD	492	CYP2E1	AD-PD
220	PCDH11X	AD	493	GAK	AD-PD
221	APH1B	AD	494	FGB	AD-PD
222	ADAMTS1	AD	495	TMEM230	AD-PD
223	DDIT4	AD	496	VDR	AD-PD
224	SLC2A4	AD	497	ESR2	AD-PD
225	ENO2	AD	498	HLA-DRB1	AD-PD
226	IGF2R	AD	499	PLA2G6	AD-PD
227	GSTA4	AD	500	CASP3	AD-PD
228	LIPC	AD	501	GSTT1	AD-PD
229	ADRA1A	AD	502	GIGYF2	AD-PD
230	UQCRC1	AD	503	IGF1R	AD-PD
231	MAPT-AS1	AD	504	MTHFR	AD-PD
232	ATM	AD	505	TRPM2	AD-PD
233	PIK3R1	AD	506	NQO2	AD-PD
234	CASP7	AD	507	GSTO1	AD-PD
235	DLST	AD	508	KLK6	AD-PD
236	KCNMA1	AD	509	GSTO2	AD-PD
237	PON2	AD	510	GDF5	AD-PD
238	PCK1	AD	511	CASP9	AD-PD
239	PPP3R1	AD	512	CREB1	AD-PD
240	APBB2	AD	513	EPHX1	AD-PD
241	GSTM3	AD	514	CAST	AD-PD
242	IREB2	AD	515	BAG5	AD-PD
243	MBL2	AD	516	MAP3K5	AD-PD
244	WT1	AD	517	HGF	AD-PD
245	CDK5R1	AD	518	INSR	AD-PD
246	NEFM	AD	519	DDIT4	AD-PD
247	NDUFV2	AD	520	ENO2	AD-PD
248	RFC1	AD	521	IGF2R	AD-PD
249	VCP	AD	522	GSTA4	AD-PD
250	TNR	AD	523	MAPT-AS1	AD-PD

251	MIR181C	AD	524	HMGCR	AD-PD
252	SLC30A4	AD	525	ATM	AD-PD
253	HBG1	AD	526	A2M	AD-PD
254	MAP2	AD	527	NDUFV2	AD-PD
255	TPI1	AD	528	EIF2AK2	AD-PD
256	PGRMC1	AD	529	RFC1	AD-PD
257	SLC30A6	AD	530	TNR	AD-PD
258	AIF1	AD	531	MIR181C	AD-PD
259	PYY	AD	532	HBG1	AD-PD
260	ADRB3	AD	533	MAP2	AD-PD
261	SPRED2	AD	534	AIF1	AD-PD
262	TPH1	AD	535	MTRR	AD-PD
263	TAP2	AD	536	PHACTR2	AD-PD
264	MCM2	AD	537	CNTNAP2	AD-PD
265	MTRR	AD	538	MAG	AD-PD
266	PHACTR2	AD	539	EDN1	AD-PD
267	UNC13C	AD	540	ADARB2	AD-PD
268	CCDC6	AD	541	TCN2	AD-PD
269	CNTNAP2	AD	542	FBP1	AD-PD
270	MIR100	AD	543	SLC30A10	AD-PD
271	MAG	AD	544	RPL6	AD-PD
272	EDN1	AD	545	SLC38A2	AD-PD
273	ADARB2	AD	546	PTGIS	AD-PD

Table S4. Molecular docking of ACEA phytocompounds with TNF-α.

NO.	PubChem ID	Ligands	Docking score (kcal/mol)
	3152	Donepezil<br>(AD drug - reference ligand)	-6.192
	119570	Pramipexole<br>(PD drug - reference ligand)	-4.602
	26757	Selegiline<br>(PD drug - reference ligand)	-4.182
	16079006	SPD-304<br>(positive control)	-6.9
1	156059	Lucidine B	-7.965
2	82143	Cryptolepine	-7.262
3	73419	Solanocapsine	-7.261
4	441082	Conessine	-6.931
5	442952	Dimethamine	-6.811
6	441663	Silandrin	-6.763

7	442493	Oxolucidine B	-6.554
8	98285	1,2,3,4-Tetrahydro-Beta-Carboline-3-Carboxylic Acid	-6.408
9	94391	Dehydroabietic acid	-6.371
10	73062	Kaurenoic Acid	-6.342
11	3081405	Phellodendrine	-6.33
12	441831	Podecdysone B	-6.282
13	73117	(+)-Eudesmin	-6.189
14	441960	Cimifugin	-6.098
15	442037	Carapanaubine	-6.053
16	73588	Taxodione	-6.031
17	92112	Eburnamonine	-6.002
18	443690	Vasconine	-5.976
19	439652	(2S)-Flavanone	-5.975
20	68230	Flindersine	-5.942
21	6442617	Dehydrocurdione	-5.822
22	94151	Cinchonamine	-5.809
23	46174050	Dihydroshikonofuran	-5.7
24	5281559	senkyunolide B	-5.658
25	11953925	Montanol	-5.501
26	10178	Tetryl	-5.403
27	5281514	Buddledin A	-5.278
28	5961	L-Glutamine	-5.04
29	78673	Tremetone	-4.875
30	6508	Quinic acid	-4.862
31	122841	Aspidinol	-4.75
32	191120	Erythrocentaurin	-4.64
33	11127215	Ecgonone methyl ester	-4.346
34	72386	Thiarubrine A	-4.308
35	122121	Phytosphingosine	-4.277
36	439700	Methylitaconate	-4.087
37	441447	Homostachydrine	-4.071
38	473	4-Methylthio-2-oxobutanoic acid	-3.562
39	91486	Sphinganine	-3.349
40	5192	Sebacic acid	-3.189
41	12253	N-Ethylacetamide	-3.015
42	166766	Lathyrine	Not docked #

Compound docking was not processed due to being unable to fit the binding pocket of the protein.

Table S5. Molecular docking of ACEE phytocompounds with TNF-α.

NO.	PubChem ID	Ligands	Docking score (kcal/mol)
	3152	Donepezil (AD drug - reference ligand)	-6.192
	119570	Pramipexole (PD drug - reference ligand)	-4.602
	26757	Selegiline (PD drug - reference ligand)	-4.182
	16079006	SPD-304 (positive control)	-6.9
1	442088	Evodiamine	-7.039
2	5281266	Cassaidine	-6.684
3	442190	Cassyfiline	-6.54
4	441831	Podecdysone B	-6.282
5	73117	(+)-Eudesmin	-6.189
6	114829	Liquiritigenin	-6.07
7	73620	Crinamine	-5.766
8	189061	Procurcumenol	-5.713
9	3037048	Isorhynchophylline	-5.515
10	11953925	Montanol	-5.501
11	442870	Pithecolobine	-5.456
12	5281514	Buddledin A	-5.278
13	69188	Ethyl nicotinate	-5.088
14	6508	Quinic acid	-4.862
15	5570	Trigonelline	-3.867
16	5281119	Myristoleic acid	-3.814
17	115244	Stachydrine	-3.61

Table S6. Molecular docking of ACEA compounds with IL-1β.

NO.	PubChem ID	Ligands	Docking score (kcal/mol)
	3152	Donepezil (AD drug - reference ligand)	-6.934
	119570	Pramipexole (PD drug - reference ligand)	-4.792
	26757	Selegiline (PD drug - reference ligand)	-4.568

	5280343	Quercetin (positive control)	-8.2
1	73419	Solanocapsine	-9.361
2	156059	Lucidine B	-8.713
3	441663	Silandrin	-8.694
4	442493	Oxolucidine B	-8.378
5	441082	Conessine	-8.112
6	94391	Dehydroabietic acid	-7.816
7	92112	Eburnamonine	-7.766
8	3081405	Phellodendrine	-7.716
9	441831	Podecdysone B	-7.654
10	441960	Cimifugin	-7.405
11	73062	Kaurenoic Acid	-7.349
12	73117	(+)-Eudesmin	-6.962
13	439652	(2S)-Flavanone	-6.958
14	73588	Taxodione	-6.953
15	442952	Dimethamine	-6.91
16	94151	Cinchonamine	-6.859
17	82143	Cryptolepine	-6.73
18	68230	Flindersine	-6.684
19	443690	Vasconine	-6.664
20	46174050	Dihydroshikonofuran	-6.389
21	442037	Carapanaubine	-6.298
22	98285	1,2,3,4-Tetrahydro-Beta-Carboline-3-Carboxylic Acid	-6.2
23	6442617	Dehydrocurdione	-6.171
24	11953925	Montanol	-6.024
25	10178	Tetryl	-5.941
26	78673	Tremetone	-5.762
27	6508	Quinic acid	-5.742
28	5281559	Senkyunolide B	-5.694
29	5281514	Buddledin A	-5.595
30	191120	Erythrocentaurin	-5.282
31	11127215	Ecgonone methyl ester	-5.131
32	122841	Aspidinol	-5.108
33	72386	Thiarubrine A	-5.045
34	122121	Phytosphingosine	-4.841
35	441447	Homostachydrine	-4.395

36	5961	L-Glutamine	-4.289
37	439700	Methylitaconate	-4.272
38	91486	Sphinganine	-4.036
39	5192	Sebacic acid	-3.94
40	473	4-Methylthio-2-oxobutanoic acid	-3.637
41	12253	N-Ethylacetamide	-3.545
42	166766	Lathyrine	Not docked #

Compound docking was not processed due to being unable to fit the binding pocket of the protein.

Table S7. Molecular docking of ACEE compounds with IL-1β.

NO	PubChem ID	Ligands	Docking Score (kcal/mol)
	3152	Donepezil (AD drug - reference ligand)	-6.934
	119570	Pramipexole (PD drug - reference ligand)	-4.792
	26757	Selegiline (PD drug - reference ligand)	-4.568
	5280343	Quercetin (positive control)	-8.2
1	442088	Evodiamine	-7.897
2	441831	Podecdysone B	-7.654
3	189061	Procurcumenol	-7.205
4	73117	(+)-Eudesmin	-6.962
5	114829	Liquiritigenin	-6.855
6	442190	Cassyfiline	-6.793
7	73620	Crinamine	-6.565
8	5281266	Cassaidine	-6.543
9	442870	Pithecolobine	-6.356
10	6508	Quinic acid	-5.742
11	11953925	Montanol	-6.024
12	3037048	Isorhynchophylline	-5.835
13	5281514	Buddledin A	-5.595
14	5570	Trigonelline	-4.52
15	5281119	Myristoleic acid	-4.336
16	115244	Stachydrine	-3.993
17	69188	Ethyl nicotinate	-3.94

Table S8. Molecular docking of ACEA compounds with IL-6.

NO.	PubChem ID	Ligands	Docking score (kcal/mol)
	3152	Donepezil<br>(AD drug - reference ligand)	-5.846
	26757	Selegiline<br>(PD drug - reference ligand)	-4.551
	119570	Pramipexole<br>(PD drug - reference ligand)	-4.254
	5280343	Quercetin<br>(positive control)	-8.5
1	73419	Solanocapsine	-7.417
2	94391	Dehydroabietic acid	-6.979
3	441663	Silandrin	-6.937
4	441082	Conessine	-6.717
5	46174050	Dihydroshikonofuran	-6.699
6	441831	Podecdysone B	-6.584
7	5281559	senkyunolide B	-6.397
8	82143	Cryptolepine	-6.346
9	443690	Vasconine	-6.324
10	439652	(2S)-Flavanone	-6.308
11	92112	Eburnamonine	-6.255
12	73117	(+)-Eudesmin	-6.144
13	94151	Cinchonamine	-6.135
14	3081405	Phellodendrine	-6.123
15	442952	Dimethamine	-6.066
16	442493	Oxolucidine B	-6.035
17	73588	Taxodione	-6.02
18	68230	Flindersine	-6.008
19	73062	Kaurenoic Acid	-5.942
20	10178	Tetryl	-5.861
21	11953925	Montanol	-5.781
22	98285	1,2,3,4-Tetrahydro-Beta-Carboline-3-Carboxylic Acid	-5.752
23	441960	Cimifugin	-5.687
24	156059	Lucidine B	-5.599
25	439700	Methylitaconate	-5.352
26	6442617	Dehydrocurdione	-5.336
27	5281514	Buddledin A	-5.2

28	442037	Carapanaubine	-5.167
29	191120	Erythrocentaurin	-5.15
30	78673	Tremetone	-4.929
31	11127215	Ecgonone methyl ester	-4.85
32	6508	Quinic acid	-4.841
33	122841	Aspidinol	-4.731
34	441447	Homostachydrine	-4.403
35	5192	Sebacic acid	-4.284
36	72386	Thiarubrine A	-4.163
37	473	4-Methylthio-2-oxobutanoic acid	-3.583
38	122121	Phytosphingosine	-3.48
39	5961	L-Glutamine	-3.469
40	91486	Sphinganine	-3.337
41	12253	N-Ethylacetamide	-2.867
42	166766	Lathyrine	Not docked #

Compound docking was not processed due to being unable to fit the binding pocket of the protein.

Table S9. Molecular docking of ACEE phytocompounds with IL-6.

NO	PubChem ID	Ligands	Docking score (kcal/mol)
	3152	Donepezil (AD drug - reference ligand)	-5.846
	26757	Selegiline (PD drug - reference ligand)	-4.551
	119570	Pramipexole (PD drug - reference ligand)	-4.254
	5280343	Quercetin (positive control)	-8.5
1	442088	Evodiamine	-7.251
2	114829	Liquiritigenin	-6.905
3	441831	Podecdysone B	-6.584
4	442190	Cassyfiline	-6.262
5	5281266	Cassaidine	-6.248
6	73117	(+)-Eudesmin	-6.144
7	3037048	Isorhynchophylline	-5.939
8	11953925	Montanol	-5.781
9	73620	Crinamine	-5.574
10	189061	Procurcumenol	-5.331
11	442870	Pithecolobine	-5.255

12	5281514	Buddledin A	-5.2
13	6508	Quinic acid	-4.841
14	69188	Ethyl nicotinate	-4.742
15	5570	Trigonelline	-4.547
16	115244	Stachydrine	-3.925
17	5281119	Myristoleic acid	-3.836

Table S10. Molecular docking of ACEA phytocompounds with CASP3.

NO	PubChem ID	Ligands	Docking score (kcal/mol)
	3152	Donepezil (AD drug - reference ligand)	-6.302
	26757	Selegiline (PD drug - reference ligand)	-5.538
	119570	Pramipexole (PD drug - reference ligand)	-5.071
	12035	N-acetylcysteine (positive control)	-4.4
1	156059	Lucidine B	-8.176
2	442493	Oxolucidine B	-8.129
3	73419	Solanocapsine	-8.119
4	94391	Dehydroabietic acid	-7.604
5	439652	(2S)-Flavanone	-7.397
6	3081405	Phellodendrine	-7.243
7	73062	Kaurenoic Acid	-7.242
8	441082	Conessine	-7.173
9	441663	Silandrin	-7.056
10	441831	Podecdysone B	-6.947
11	442952	Dimethamine	-6.94
12	92112	Eburnamonine	-6.819
13	46174050	Dihydroshikonofuran	-6.582
14	82143	Cryptolepine	-6.552
15	73588	Taxodione	-6.402
16	441960	Cimifugin	-6.39
17	73117	(+)-Eudesmin	-6.366
18	5281559	Senkyunolide B	-6.33
19	443690	Vasconine	-6.12
20	98285	1,2,3,4-Tetrahydro-Beta-Carboline-3-Carboxylic Acid	-6.062

21	11953925	Montanol	-5.922
22	68230	Flindersine	-5.857
23	94151	Cinchonamine	-5.843
24	442037	Carapanaubine	-5.48
25	6442617	Dehydrocurdione	-5.345
26	191120	Erythrocentaurin	-5.295
27	11127215	Ecgonone methyl ester	-5.258
28	122841	Aspidinol	-5.231
29	6508	Quinic acid	-5.222
30	91486	Sphinganine	-5.115
31	78673	Tremetone	-5.044
32	10178	Tetryl	-4.827
33	5281514	Buddledin A	-4.808
34	441447	Homostachydrine	-4.733
35	122121	Phytosphingosine	-4.7
36	72386	Thiarubrine A	-4.591
37	439700	Methylitaconate	-4.537
38	5961	L-Glutamine	-4.526
39	5192	Sebacic acid	-4.483
40	473	4-Methylthio-2-oxobutanoic acid	-3.754
41	12253	N-Ethylacetamide	-3.449
42	166766	Lathyrine	Not docked #

Compound docking was not processed due to being unable to fit the binding pocket of the protein.

Table S11. Molecular docking of ACEE phytocompounds with CASP3.

NO	PubChem ID	Ligands	Docking Score (kcal/mol)
	3152	Donepezil (AD drug - reference ligand)	-6.302
	26757	Selegiline (PD drug - reference ligand)	-5.538
	119570	Pramipexole (PD drug - reference ligand)	-5.071
	12035	N-acetylcysteine (positive control)	-4.4
1	442088	Evodiamine	-8.948
2	3037048	Isorhynchophylline	-7.596
3	5281266	Cassaidine	-7.312
4	441831	Podecdysone B	-6.947

5	442190	Cassyfiline	-6.785
6	114829	Liquiritigenin	-6.784
7	442870	Pithecolobine	-6.754
8	73117	(+)-Eudesmin	-6.366
9	5281559	Crinamine	-6.315
10	11953925	Montanol	-5.922
11	189061	Procurcumenol	-5.787
12	6508	Quinic acid	-5.222
13	5281514	Buddledin A	-4.808
14	5570	Trigonelline	-4.503
15	5281119	Myristoleic acid	-4.235
16	69188	Ethyl nicotinate	-4.077
17	115244	Stachydrine	-4.045

Table S12. Molecular docking of ACEA phytocompounds with MAPK3.

NO.	PubChem ID	Ligands	Docking score (kcal/mol)
	3152	Donepezil (AD drug - reference ligand)	-6.79
	119570	Pramipexole (PD drug - reference ligand)	-5.5
	26757	Selegiline (PD drug - reference ligand)	-4.91
	11719003	Ulixertinib (positive control)	-6.6
1	441663	Silandrin	-8.929
2	442493	Oxolucidine B	-8.221
3	73419	Solanocapsine	-8.211
4	441082	Conessine	-8.105
5	3081405	Phellodendrine	-7.877
6	68230	Flindersine	-7.797
7	442952	Dimethamine	-7.743
8	94391	Dehydroabietic acid	-7.67
9	443690	Vasconine	-7.625
10	92112	Eburnamonine	-7.55
11	441831	Podecdysone B	-7.549
12	82143	Cryptolepine	-7.546
13	439652	(2S)-Flavanone	-7.536

14	98285	1,2,3,4-Tetrahydro-Beta-Carboline-3-Carboxylic Acid	-7.453
15	441960	Cimifugin	-7.398
16	73117	(+)-Eudesmin	-7.273
17	46174050	Dihydroshikonofuran	-7.024
18	73588	Taxodione	-6.993
19	156059	Lucidine B	-6.871
20	94151	Cinchonamine	-6.857
21	6442617	Dehydrocurdione	-6.8
22	73062	Kaurenoic Acid	-6.795
23	5281559	Senkyunolide B	-6.626
24	442037	Carapanaubine	-6.553
25	5281514	Buddledin A	-6.041
26	78673	Tremetone	-5.705
27	11953925	Montanol	-5.698
28	72386	Thiarubrine A	-5.569
29	10178	Tetryl	-5.473
30	6508	Quinic acid	-5.465
31	5192	Sebacic acid	-5.333
32	191120	Erythrocentaurin	-5.279
33	122841	Aspidinol	-5.222
34	11127215	Ecgonone methyl ester	-5.093
35	91486	Sphinganine	-4.756
36	441447	Homostachydrine	-4.67
37	5961	L-Glutamine	-4.626
38	439700	Methylitaconate	-4.588
39	122121	Phytosphingosine	-4.174
40	473	4-Methylthio-2-oxobutanoic acid	-3.598
41	12253	N-Ethylacetamide	-3.338
42	166766	Lathyrine	Not docked #

Compound docking was not processed due to being unable to fit the binding pocket of the protein.

Table S13. Molecular docking of ACEE phytocompounds with MAPK3.

NO.	PubChem ID	Ligands	Docking score (kcal/mol)
	3152	Donepezil (AD drug - reference ligand)	-6.79
	119570	Pramipexole	-5.5

		(PD drug - reference ligand)	
	26757	Selegiline<br>(PD drug - reference ligand)	-4.91
	11719003	Ulixertinib<br>(positive control)	-6.6
1	114829	Liquiritigenin	-8.151
2	441831	Podecdysone B	-7.549
3	442088	Evodiamine	-7.529
4	73117	(+)-Eudesmin	-7.273
5	5281266	Cassaidine	-6.784
6	73620	Crinamine	-6.585
7	442190	Cassyfiline	-6.338
8	189061	Procurcumenol	-6.28
9	442870	Pithecolobine	-6.207
10	5281514	Buddledin A	-6.041
11	3037048	Isorhynchophylline	-5.972
12	11953925	Montanol	-5.698
13	6508	Quinic acid	-5.465
14	5570	Trigonelline	-4.785
15	5281119	Myristoleic acid	-4.61
16	115244	Stachydrine	-4.12
17	69188	Ethyl nicotinate	-3.97

Table S14. Molecular docking of ACEA compounds with AKT1.

NO.	PubChem ID	Ligands	Docking score (kcal/mol)
	3152	Donepezil<br>(AD drug - reference ligand)	-6.92
	26757	Selegiline<br>(PD drug - reference ligand)	-5.671
	119570	Pramipexole<br>(PD drug - reference ligand)	-5.354
	25227436	Ipatasertib<br>(positive control)	-8.3
1	442493	Oxolucidine B	-9.222
2	156059	Lucidine B	-8.959
3	73419	Solanocapsine	-8.89
4	94391	Dehydroabietic acid	-8.885
5	92112	Eburnamonine	-8.777

6	82143	Cryptolepine	-8.402
7	439652	(2S)-Flavanone	-8.1
8	442952	Dimethamine	-8.018
9	441082	Conessine	-7.91
10	441663	Silandrin	-7.894
11	3081405	Phellodendrine	-7.522
12	94151	Cinchonamine	-7.337
13	442037	Carapanaubine	-7.324
14	68230	Flindersine	-7.295
15	73062	Kaurenoic Acid	-7.29
16	441831	Podecdysone B	-7.126
17	73117	(+)-Eudesmin	-7.113
18	6442617	Dehydrocurdione	-6.944
19	5281559	senkyunolide B	-6.823
20	98285	1,2,3,4-Tetrahydro-Beta-Carboline-3-Carboxylic Acid	-6.808
21	46174050	Dihydroshikonofuran	-6.732
22	73588	Taxodione	-6.614
23	78673	Tremetone	-6.446
24	443690	Vasconine	-6.357
25	191120	Erythrocentaurin	-6.237
26	11953925	Montanol	-6.149
27	11127215	Ecgonone methyl ester	-5.963
28	10178	Tetryl	-5.712
29	441960	Cimifugin	-5.594
30	122841	Aspidinol	-5.557
31	6508	Quinic acid	-5.41
32	5281514	Buddledin A	-5.255
33	72386	Thiarubrine A	-5.195
34	441447	Homostachydrine	-4.96
35	5192	Sebacic acid	-4.815
36	122121	Phytosphingosine	-4.686
37	5961	L-Glutamine	-4.497
38	12253	N-Ethylacetamide	-4.324
39	439700	Methylitaconate	-4.22
40	91486	Sphinganine	-4.201
41	473	4-Methylthio-2-oxobutanoic acid	-3.879
42	166766	Lathyrine	Not docked [#]

Compound docking was not processed due to being unable to fit the binding pocket of the protein.

Table S15. Molecular docking of ACEE phytocompounds with AKT1.

NO	PubChem ID	Ligands	Docking score (kcal/mol)
	3152	Donepezil (AD drug - reference ligand)	-6.92
	26757	Selegiline (PD drug - reference ligand)	-5.671
	119570	Pramipexole (PD drug - reference ligand)	-5.354
	25227436	Ipatasertib (positive control)	-8.3
1	442088	Evodiamine	-7.9
2	114829	Liquiritigenin	-7.737
3	73620	Crinamine	-7.365
4	441831	Podecdysone B	-7.126
5	73117	(+)-Eudesmin	-7.113
6	189061	Procurcumenol	-6.795
7	3037048	Isorhynchophylline	-6.779
8	5281266	Cassaidine	-6.25
9	442190	Cassyfiline	-6.244
10	11953925	Montanol	-6.149
11	442870	Pithecolobine	-5.75
12	6508	Quinic acid	-5.41
13	5281514	Buddledin A	-5.255
14	69188	Ethyl nicotinate	-5.123
15	5570	Trigonelline	-4.91
16	5281119	Myristoleic acid	-4.581
17	115244	Stachydrine	-3.995

Table S16. Primer sequences used in RT-qPCR analysis.

Gene	Forward primer (5′ → 3′)	Reverse primer (5′ → 3′)
Tnfa	GATCGGTCCCCAAAGGGATG	TAGCAAATCGGCTGACGGTG
Il1b	GAAATGCCACCTTTTGACAGTG	CTGGATGCTCTCATCAGGACA
Il6	TCTTGGGACTGATGCTGGTG	CAGGTCTGTTGGGAGTGGTA
Casp3	CATGGGAGCAAGTCAGTGGA	TGTCTCTCTGAGGTTGGCTG
Mapk3	CCTGAAGCCTTCCAATCTGC	AAGCATGATCTCTGGGGCTC
Akt1	CCACGCTACTTCCTCCTCAA	AGGCAGCGGATGATAAAGGT
Actb (β-actin)	GGCTGTATTCCCCTCCATCG	CCAGTTGGTAACAATGCCATGT
